# Endogenous, tissue-resident stem/progenitor cells in gonads and bone marrow express FSHR and respond to FSH via FSHR-3

**DOI:** 10.1186/s13048-021-00883-0

**Published:** 2021-10-30

**Authors:** Deepa Bhartiya, Hiren Patel, Ankita Kaushik, Pushpa Singh, Diksha Sharma

**Affiliations:** 1 Stem Cell Biology Department, ICMR- National Institute for Research in Reproductive Health, Jehangir Merwanji Street, Parel, Mumbai, 400 012 India; 2 Present address: Department of Ophthalmology and Visual Sciences, University of Nebraska Medical Center, Omaha, NE USA

**Keywords:** FSH, FSHR, FSHR-3, Ovary, Testis, Uterus, Bone marrow, Very small embryonic-like stem cells (VSELs)

## Abstract

Follicle stimulating hormone (FSH) is secreted by the anterior pituitary and acts on the germ cells indirectly through Granulosa cells in ovaries and Sertoli cells in the testes. Extragonadal action of FSH has been reported but is still debated. Adult tissues harbor two populations of stem cells including a reserve population of primitive, small-sized, pluripotent very small embryonic-like stem cells (VSELs) and slightly bigger, tissue-specific progenitors which include ovarian stem cells (OSCs) in ovaries, spermatogonial stem cells (SSCs) in testes, endometrial stem cells (EnSCs) in uterus and hematopoietic stem cells (HSCs) in the bone marrow. Data has accumulated in animal models showing FSHR expression on both VSELs and progenitors in ovaries, testes, uterus and bone marrow and eventually gets lost as the cells differentiate further. FSH exerts a direct action on the stem/progenitor cells via alternatively spliced FSHR-3 rather than the canonical FSHR-1. FSH stimulates VSELs to undergo asymmetrical cell divisions to self-renew and give rise to the progenitors that in turn undergo symmetrical cell divisions and clonal expansions followed by differentiation into specific cell types. Excessive self-renewal of VSELs results in cancer and this explains ubiquitous expression of embryonic markers including nuclear OCT-4 along with FSHR in cancerous tissues. Focus of this review is to compile published data to support this concept. FSHR expression in stem/progenitor cells was confirmed by immuno-fluorescence, Western blotting, in situ hybridization and by quantitative RT-PCR. Two different commercially available antibodies (Abcam, Santacruz) were used to confirm specificity of FSHR expression along with omission of primary antibody and pre-incubation of antibody with immunizing peptide as negative controls. Western blotting allowed detection of alternatively spliced FSHR isoforms. Oligoprobes and primers specific for Fshr-1 and Fshr-3 were used to study these alternately-sliced isoforms by in situ hybridization and their differential expression upon FSH treatment by qRT-PCR. To conclude, stem/progenitor cells in adult tissues express FSHR and directly respond to FSH via FSHR-3. *These findings change the field of FSH-FSHR biology, call for paradigm shift, explain FSHR expression on cancer cells in multiple organs* and provide straightforward explanations for various existing conundrums including extragonadal expression of FSHR.

## Key points

- Two populations of stem cells exist in multiple adult tissues including quiescent VSELs and slightly bigger, tissue-specific progenitors including OSCs in ovaries, SSCs in testes, EnSCs in the uterus, HSCs in the bone marrow and so on in other adult organs as well. These stem/progenitor cells express FSHR which eventually gets degraded as cells differentiate further into tissue-specific mature cell types. *Existing dogma that FSH action is limited to Granulosa cells in ovaries and Sertoli cells in the testes needs an urgent revision.*
- FSH acts directly on the VSELs that exist in all adult tissues including ovaries, testes, uterus and bone marrow. FSH exerts a direct effect on the stem/progenitor cells and promotes asymmetrical, symmetrical divisions and clonal expansion. FSH effects on extragonadal tissues including uterus and bone marrow stem/progenitor cells is clearly delineated in this review.
- In addition to the existing dogma that FSH exerts an indirect action on the germ cells via Sertoli cells in testes or granulosa cells in the ovaries, FSH directly affects testicular and ovarian stem cells.
- FSH action on the stem/progenitor cells is mediated via Fshr-3 which is alternately spliced, growth factor type-1 receptor that acts via calcium signaling and the ERK/MAPK pathway.
- Stem cells are involved in both endometrial regeneration and hematopoiesis in bone marrow and FSH has a potential role in both these processes.
- Expression of FSHR on VSELs and OSCs in the ovaries explains why commercially available ovarian cancer cell lines when treated with FSH do not show increase in cAMP. Gonadotropin theory with a potential role of stem cells in the OSE could possibly explain initiation of ovarian cancer.
- Our findings explain extragonadal expression of FSHR in the uterus. FSH exerts direct action on both endometrial and myometrial stem cells. Various uteropathies have a stem cell basis and this explains FSHR expression in clinical samples of endometriosis, endometrial cancers as well as myomas.
- Several folds increased Fshr-3 in testicular cancer samples is intriguing and suggests a role of FSH in initiating testicular cancer similar to gonadotropin theory that exists for ovarian cancer.

## Main text

It is textbook information, after almost 90 years of research since follicle-stimulating hormone (FSH) was first reported in 1930s, that FSH is secreted by the anterior pituitary and acts on granulosa cells in the ovarian pre-antral follicles and on Sertoli cells in the testes [1]. It facilitates follicular growth and spermatogenesis and thus plays a central role in mammalian reproduction. FSH is critical for preantral to later-stage follicle development, whereas early-stage follicle development (primordial to primary stages) is thought to be independent of FSH action. FSH exerts an indirect effect on the germ cells in the testes by stimulating Sertoli cells to secrete growth factors required for germ cells proliferation/differentiation. Besides physiologic, FSH also exerts therapeutic effect on the gonads and is used in the ART Clinics to stimulate the ovaries to generate multiple eggs for infertile couples and in men with hypogonadotropic hypogonadism. In recent times, it has also been suggested to treat men with idiopathic infertility with FSH and to evolve ‘testicular hyperstimulation’ regimens [2, 3]. Published reports regarding FSHR expression on multiple extra-gonadal adult tissues and also on cancer cells affecting multiple organs has raised many eyebrows [4] and we have recently addressed various concerns [5].

Present review is a compilation of the work published since 2013 suggesting that FSHR are expressed on the tissue-resident stem/progenitor cells in multiple organs including bone marrow and reproductive tissues ovaries, testes and uterus. These results call for a paradigm shift in the field since evidently FSH exerts a direct action on the stem cells via Fshr-3 rather than the canonical Fshr-1 isoform. Tissue-resident stem cells that exist in adult tissues are the very small embryonic-like stem cells (VSELs) and were recently reviewed [6–8]. FSHR expression on stem/progenitor cells completely renovates the field of FSH/FSHR biology and provides straightforward explanations for various existing conundrums in the field including extragonadal expression of FSHR [1, 5, 9–11]. FSHR expression is reported on murine embryonic stem (mES) cell line (ES-D3) and teratocarcinoma cell lines P19 and NTera2 [12]. A scRNAseq study has reported low-level expression of FSHR transcripts only in female FGC 4 (oogenesis) and male FGC 3 (mitotic arrest) [13] rather than in the somatic cells in gonadal ridges. VSELs are developmentally linked to primordial germ cells [7] and both mES cells and teratocarcinoma cell lines (more primitive to primordial germ cells) also express FSHR. Thus, FSHR expression on VSELs is along the expected lines and should not surprise the readers.

### Ovarian stem cells

It is widely believed and textbook information that FSH acts on Granulosa cells in the ovaries and Sertoli cells in the testes [1]. However, besides granulosa cells, FSHR expression has also been reported on ovary surface epithelial (OSE) cells by several groups. Also, Fshr-3 was suggested to be the predominant isoform in sheep ovarian follicles. More than 90% of ovarian cancers initiate in the OSE however, OSE cells remain poorly studied till date. Alternately spliced FSHR isoforms have been reported in clinical samples, ovarian cancers and also on serous ovarian cell lines [14–16]. Contradictory views exist regarding the role of FSH in initiating ovarian cancer. FORKO mice develop ovarian cancers despite complete elimination of FSHR and lack of ovulation [17]. Another group reported that over-expression of FSHR is associated with development of ovarian epithelial cancer [18]. Li et al. [19] showed that rather than the canonical FSHR-1, alternately spliced FSHR-3 isoform mediated signaling promoted proliferation of mouse tumorigenic OSE cells ID8 by using multiple evidences at DNA, RNA and protein levels (Table 1).

**Table 1:** Result highlights of the study published by Li et al. [18] showing tumorigenic ID8 ovary cancer surface epithelial cells are affected by FSH via FSHR-3

Experiments	Results highlights
In vitro studies	ID8, a tumorigenic cell line derived from mouse ovarian surface epithelial cells when exposed to EGF (10 ng/ml) or FSH (20 ng/ml), showed greater proliferation response after FSH treatment compared to EGF in vitro.
Associated signaling	The canonical FSHR-1 is associated with stimulation of adenylate cyclase whereas the growth factor variant FSHR-3 signals via ERK1/2 activation and calcium influx in the absence of increased intracellular concentrations of cAMP. In mammalian cells transfected with FSH-R3, FSH has been shown not only to increase the influx of extracellular calcium, but also to stimulate cell proliferation and ERK phosphorylation in a calcium-dependent fashion FSH failed to stimulate cAMP accumulation in ID8 cells despite the presence of functional adenylate cyclase. Rather, FSH stimulated ERK phosphorylation. After FSH addition, the phosphorylated forms of ERK1 and ERK2 were evident at 10 min, elevated after 30 min and declined to basal levels after 60 min. Pretreatment of cells with MEK inhibitor [PD98059, 100 μM] abolished FSH mediated proliferation as well as cell signaling Effects of calcium channel antagonist SNX-482 were assessed on ID8 MOSEC to determine FSH effect on influx of calcium via voltage-gated channels. SNX-482 prevented FSH from stimulating both MOSEC growth and ERK activation
Southern Blotting	Oligonucleotide probes specific for Exons 7 & 11 were used for Southern blot analysis of genomic DNA isolated from ID8 cells. Hybridization analysis with both the probes recognized 11.6 and 4.3 kb fragments
Northern Blotting	RNA blot using the same probes as above identified 1.9 Kb band
Western Blotting	Commercial antibody against FSHR (Santacruz) directed against an N-terminal sequence of the FSH-R recommended to detect FSHR-1 was found to detect two bands of 75 and approximately 50 kDA which corresponded to FSHR-1 and FSHR-3 respectively
Immunocytochemistry	Surface expression of FSHR-3 and SNX-482 sensitive Cav2.3 channel in MOSEC was shown by immunocytochemistry

**Fig. 1:**
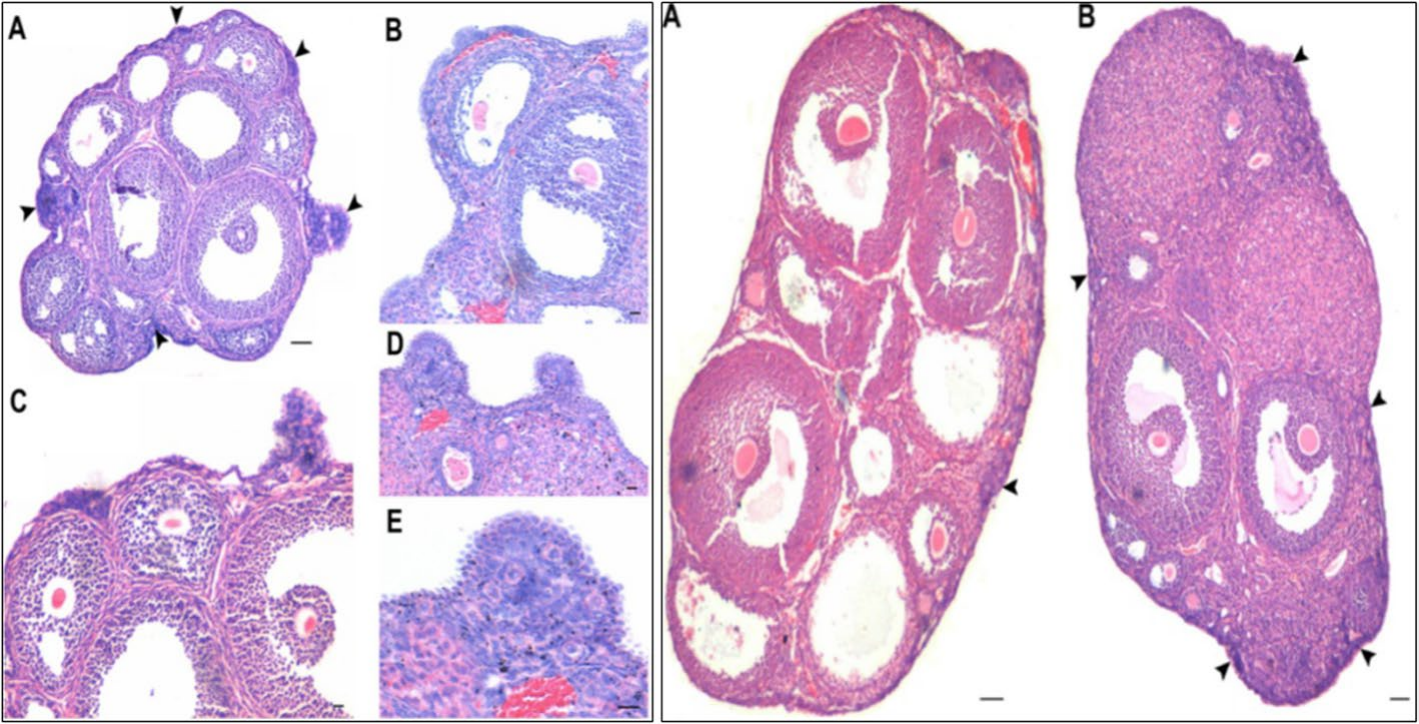
FSH acts directly on ovary surface epithelium (OSE). H&E-stained sections of mice ovaries after two (left panel) and seven (right panel) days of PMSG treatment (5 IU). Note proliferation of OSE (arrowheads) and small protuberances were prominent [Scale: A 100 μm, B-E 20 μm]. Seven days later (right panel) increased cohorts of primordial follicles were observed just below the OSE [Scale: 100 μm]. Besides stimulating follicular growth, FSH stimulated OSE cells proliferation [20]

We published an article in 2012 [20], where mice when treated with PMSG, exhibited marked proliferation of OSE cells on Day 2 and surprisingly large numbers of primordial follicles cohorts were observed in the cortical tissue on D7 after treatment (5–6 after PMSG compared to 1–2 cohorts in vehicle treated ovaries). Similar proliferation of OSE cells was also observed when mice were treated with rFSH [21]. These results of a direct action of PMSG/FSH on OSE cells intrigued us since it is widely believed that FSH acts on granulosa cells in the ovaries and that initial follicular growth is gonadotropin independent [1] (Fig. 1).

**Fig. 2:**
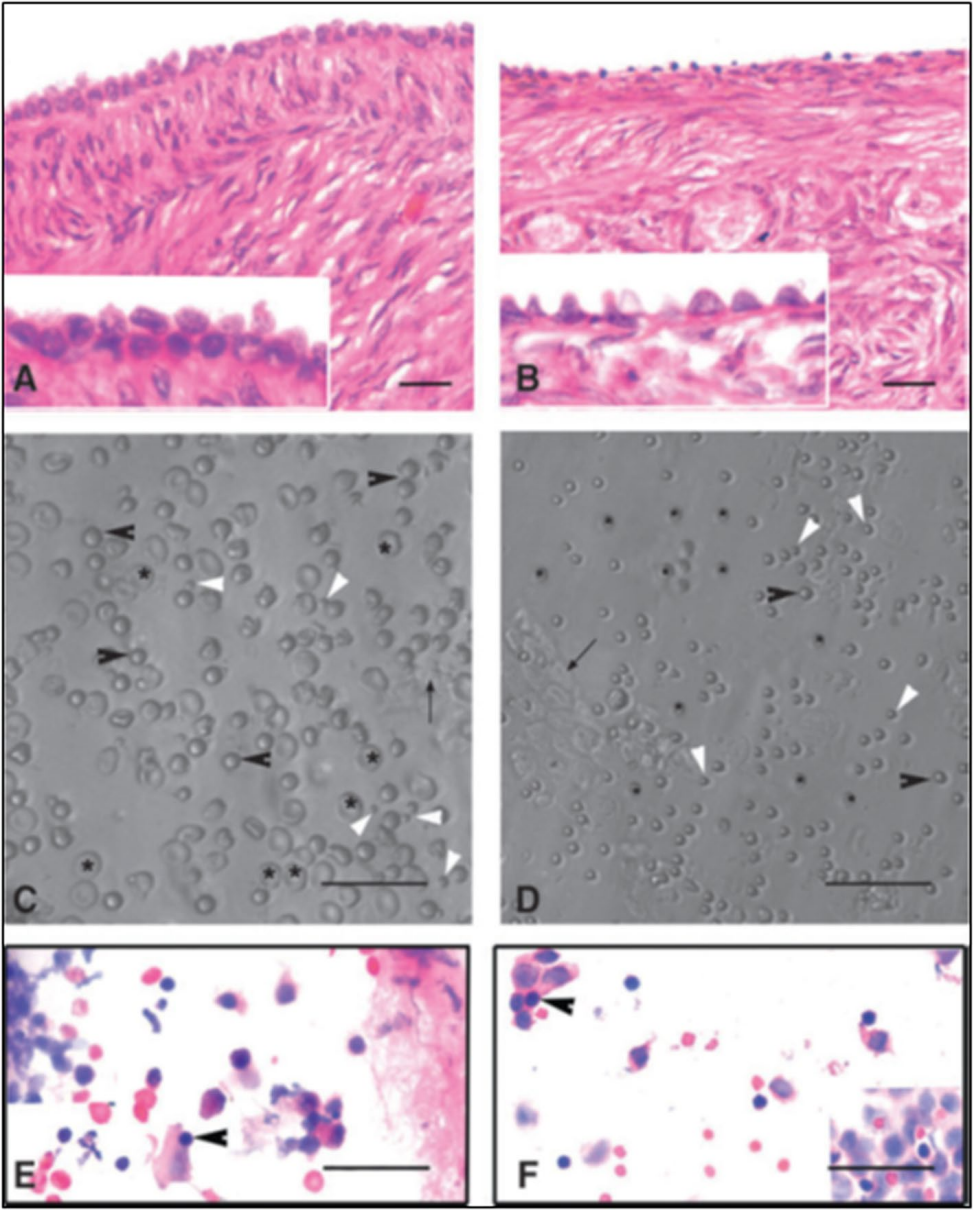
Stem cells reside in adult ovary surface epithelium (OSE) **A& B**. H&E stained sections of menopausal human (left panel) and sheep (right panel) ovarian cortex. Note prominent OSE cells which were scraped and collected in a dish. **C& D**. Note the presence of stem cells under Hoffman optics amongst OSE cells including small VSELs (white arrowhead), OSCs (black arrowhead) along with red blood cells (asterix). Epithelial cells with pale stained nuclei and abundant pink cytoplasm were also observed **E & F**. Darkly stained, spherical stem cells in H&E stained cell smears were clearly visualized (arrowheads) [23]. Scale: 20 μm

These findings of FSH action on the OSE cells become more intriguing since ovarian stem cells are reported to reside in the OSE. Presence of stem cells in adult ovaries were initially reported by Tilly’s group [22] in adult mice OSE and these stem cells can be enriched by gentle scraping of the OSE in several adult mammalian species including rabbit, marmoset, sheep, humans [23–26] and by enzymatic isolation of small-sized mice ovaries [27]. The stem cells are visualized as distinct spherical cells with high nucleo-cytoplasmic ratio with dark Hematoxylin-stained nuclei. Whereas epithelial cells are bigger in size with abundant, pink cytoplasm and pale stained nuclei (Fig. 2).

In a review describing various aspects of the ovarian stem cells [28], we have discussed that ovary harbors two populations of stem cells including 2–6 μm, very small embryonic-like stem cells (VSELs) and slightly bigger 6–8 μm ovarian stem cells (OSCs). One may refer to published literature for further details on VSELs [6–8, 26]. These stem cells differentiate in vitro into oocyte-like structures [23, 29], OSCs can be expanded over long periods in vitro and upon transplantation have resulted in live births [30–32] and also VSELs are increased in numbers in clinical samples of ovarian cancers [33–35].

**Fig. 3:**
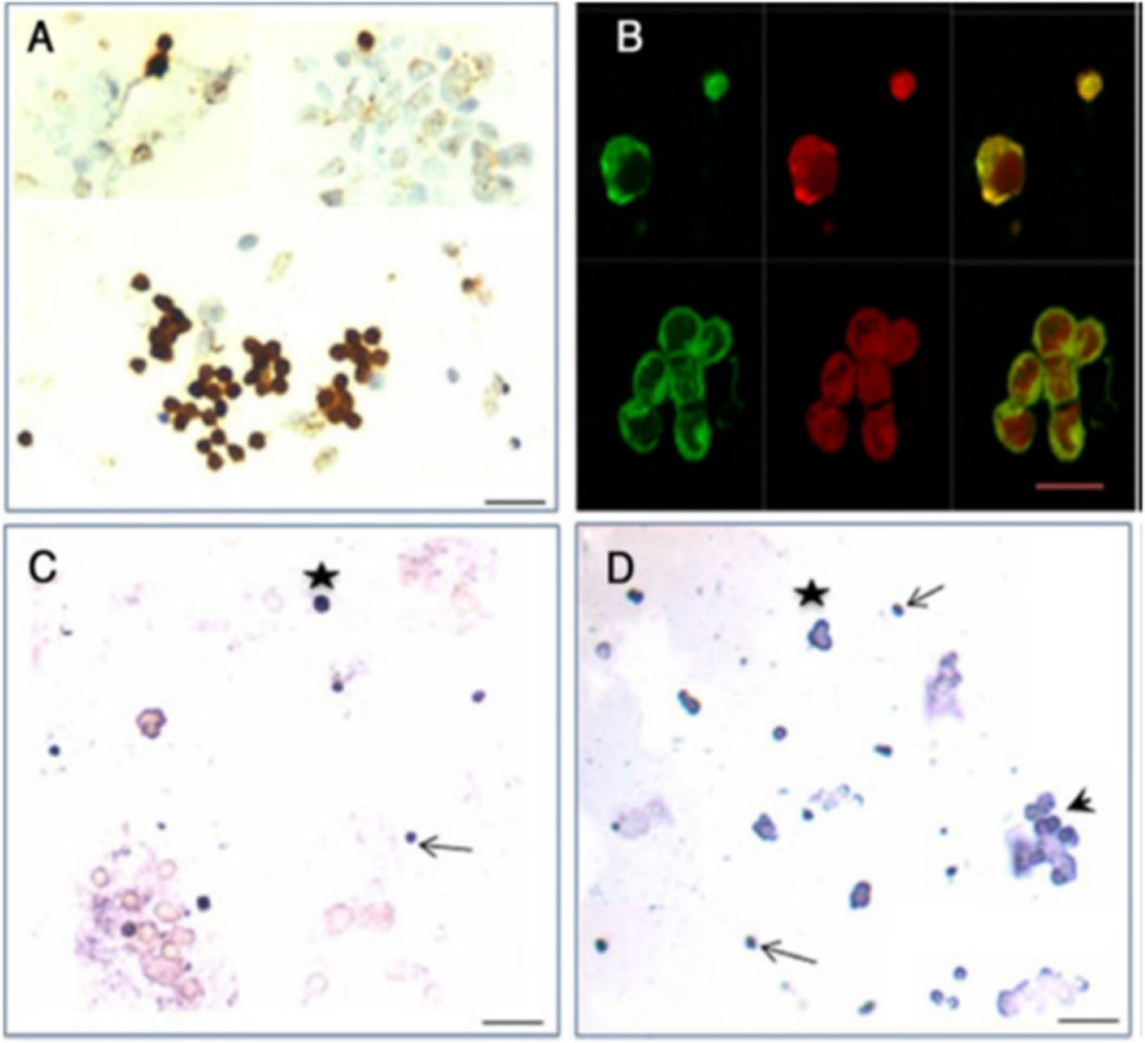
FSHR expression on ovarian stem cells. **A.** Immuno-localization studies on OSE cell smears shows that only the stem cells express FSHR and epithelial cells remain distinctly negative. **B.** Immunofluorescence studies localized FSHR in small VSELs, slightly bigger OSCs and in a cluster of cells. In situ hybridization results using specific oligo probes showed expression of (**C**) Fshr-1 and (**D**) Fshr-3 transcripts on FSH treated sheep OSE smears. As evident Fshr-1 transcripts were observed in the nuclei of both VSELs (arrow) and OGSCs (asterix). Fshr-3 transcripts were localized both in the nuclei and cytoplasm and even in the germ cell nests ‘cysts’. Presence of Fshr-3 in both cytoplasm and nuclei suggested active involvement of Fshr-3 transcript during FSH action on the stem cells [24]. Scale: 20 μm

Stem cells located in sheep OSE cells, collected by gentle scraping, were found to express FSHR (besides stem cell markers) whereas the epithelial cells remained distinctly negative (Fig. 3) [24]. Expression of FSHR exclusively on the stem cells and not on the epithelial cells was confirmed by immunocytochemistry (Fig. 3A), confocal microscopy (Fig. 3B) and by in situ hybridization (Fig. 3C, D). Evidently cancer cells in the ovary are not epithelial cells and neither arise by epithelial-mesenchymal transition as generally believed [36] but cancer represents excessive expansion of VSELs and OSCs residing in the OSE and tumorigenic ID8 cells most likely represent expanded cancer cells derived from the stem cells that reside amongst the ovary epithelial cells. The ovarian stem cells as well as the cancer cells that arise in the OSE express FSHR.

**Fig. 4:**
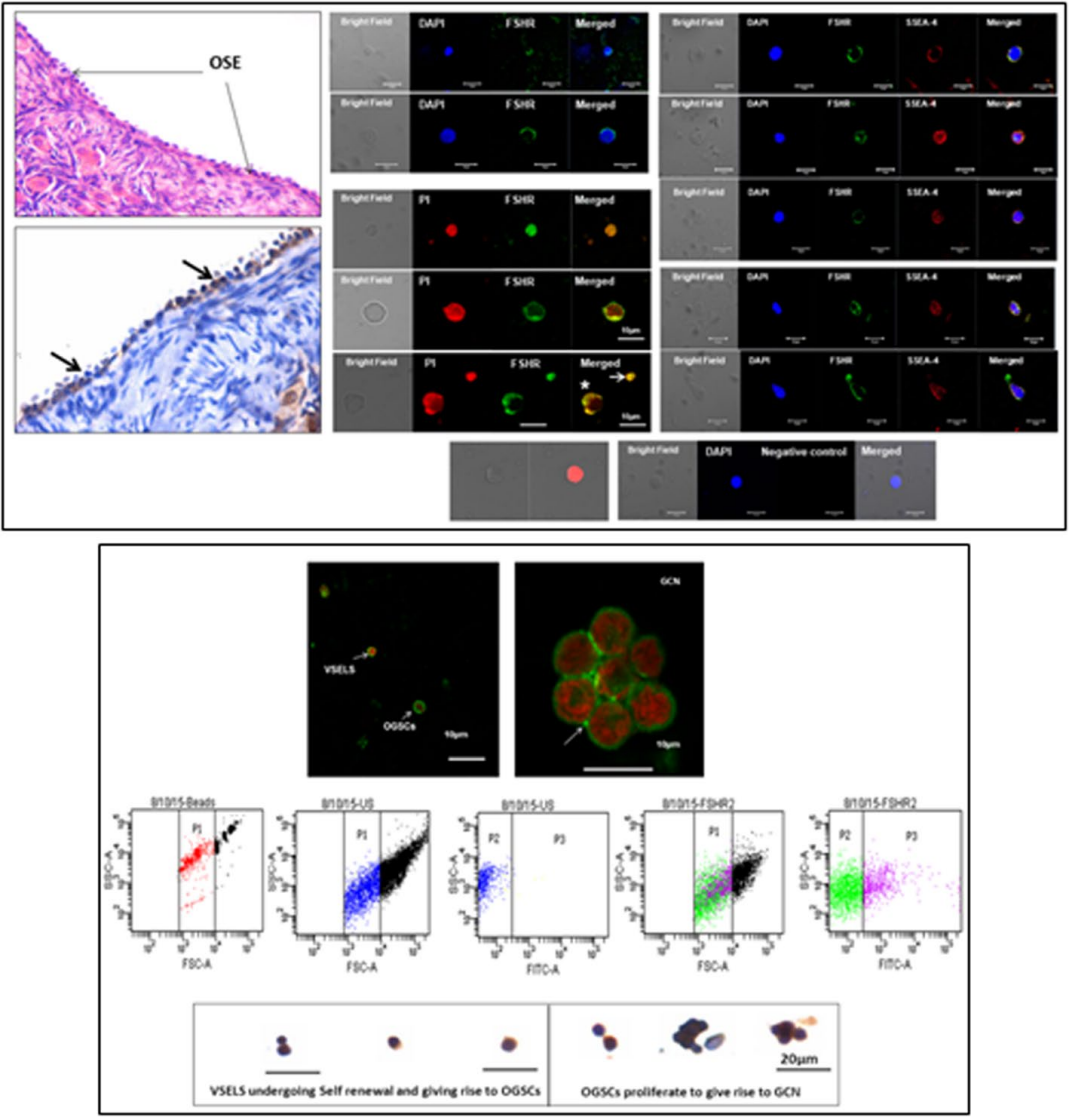
Upper panel: Sheep ovarian sections on the left clearly show FSHR expression in the OSE. Stem cells isolated from sheep OSE expressed FSHR. Co-expression of FSHR with SSEA-4 (cell surface marker for pluripotent stem cells) was clearly evident confirming FSHR expression on the stem cells. Lower panel: FSHR expression on VSELs and OSCs and on a germ cell nest (multiple cells cluster with cytoplasmic continuity). 2–6 μm cells were studied for FSHR expression by flow cytometry. Asymmetric, symmetric divisions and clonal expansion of stem cells is shown [24, 25]

Immuno-localization studies were carried out further for FSHR expression in sheep ovarian sections as well as by confocal microscopy on sheep OSE cell smears (Fig. 4). FSHR expression was observed on both VSELs and OSCs. Co-expression of FSHR was also detected with a pluripotent stem cell marker SSEA-4. Cell surface expression of FSHR was clearly observed. The stem cells were also immuno-phenotyped for FSHR by flow cytometry. Two distinct populations of stem cells were distinctly observed undergoing asymmetrical, symmetrical divisions and clonal expansion (Fig. 4 B).

**Fig. 5:**
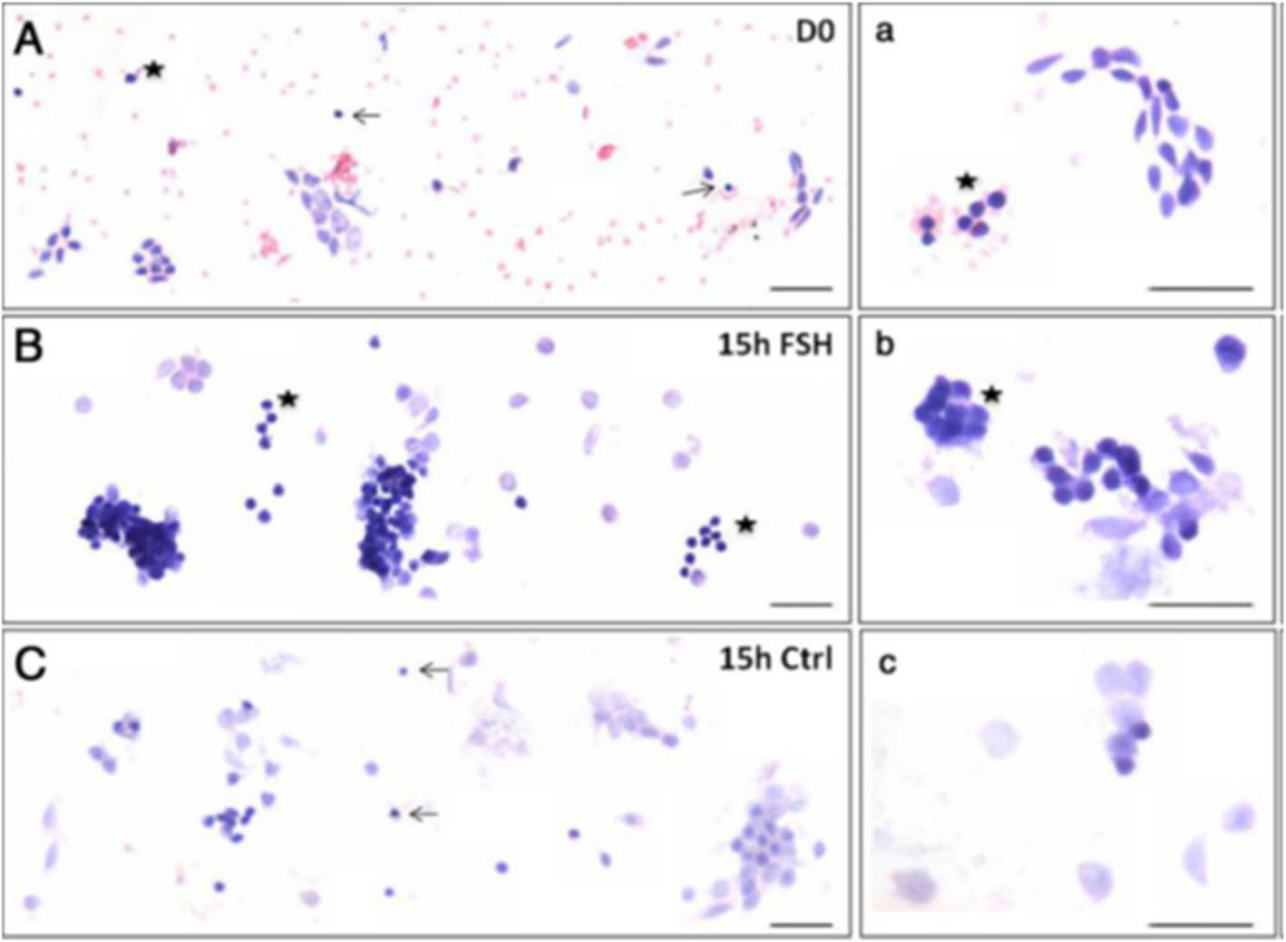
Effect of FSH treatment on the ovarian stem cells located in the ovary surface epithelium. **A&a** Freshly prepared sheep OSE smear after H & E staining. Epithelial cells (spindle-shaped cells with pale nuclei and abundant cytoplasm) and distinct populations of darkly-stained putative stem cells including the VSELs (arrow) and OSCs (asterisk) were evident along with red blood cells (RBCs) **B&b** Stem cells and germ cell ‘cysts’ were increased after 15 h of FSH treatment. The nests represent clonal expansion of stem cells with incomplete cytokinesis **C&c** OSE smear after 15 h culture without FSH. Scale: 20 μm [24]

**Fig. 6:**
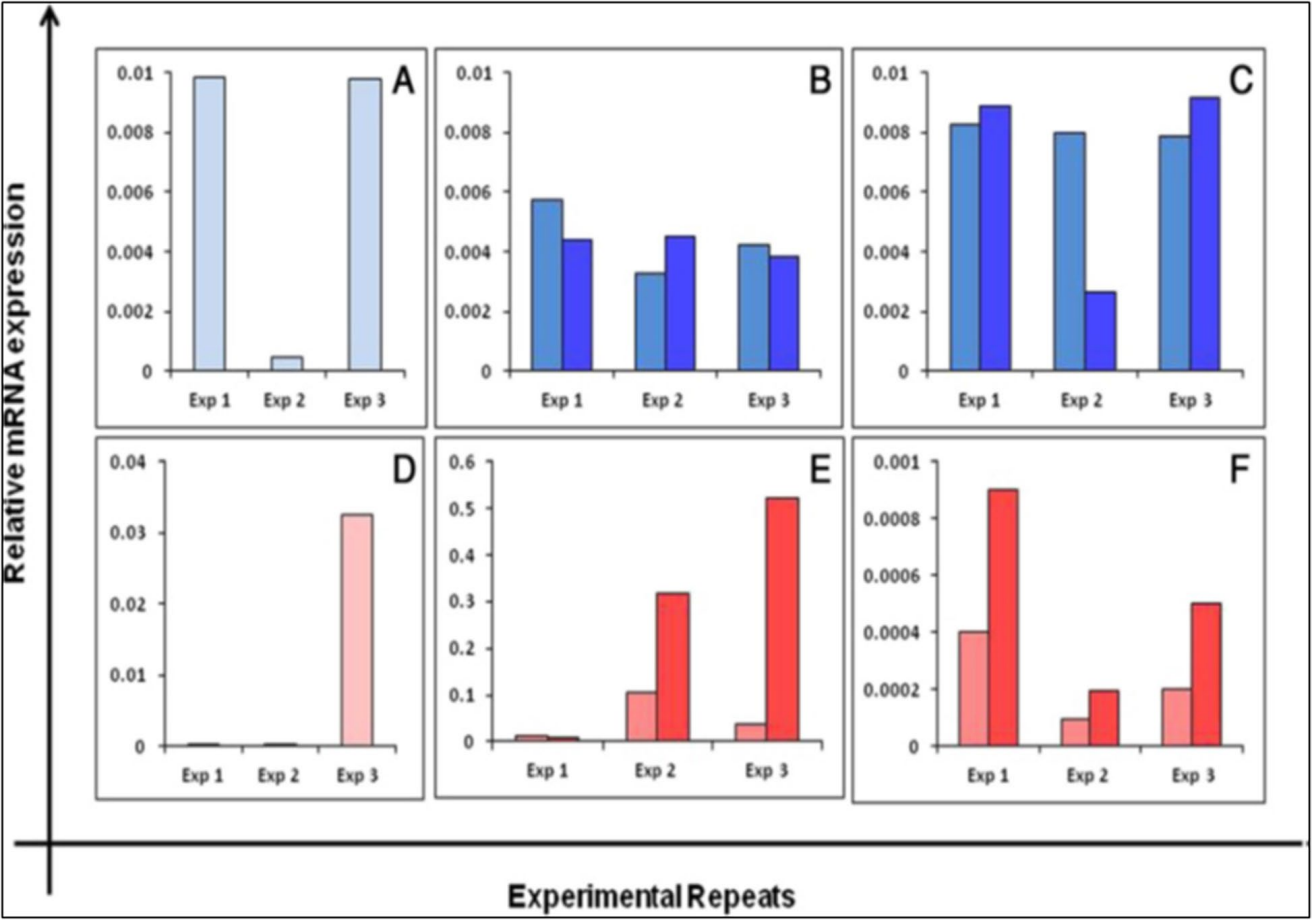
Differential expression of Fshr-1 (upper panel) and Fshr-3 (lower panel) transcripts in sheep OSE cells after treating with FSH. Fshr-1 transcripts (dark blue bars) in vitro after 0, 3 and 15 h or without (light blue bars) FSH treatment (**A**-**C**). Evidently Fshr-1 was not affected by FSH treatment. Whereas Fshr-3 after FSH (red bars) showed upregulation compared to without FSH (pink bars) treatment. Expression of Fshr-3 was maximal after 3 h (please note a change in Y-axis scale to appreciate a difference between (**D**-**F**). The relative expression levels of each transcript from three different experiments are represented individually [24]. These results of FSH action on normal sheep OSE cells via Fshr-3 in vitro are similar to those reported for tumorigenic cell line ID8 and discussed above in Table 1 [19]

Effects of treating sheep OSE cells in vitro to FSH were also studied [24]. It was observed that stem cells expanded in numbers and formed increased numbers of germ cell nests (Fig. 5). Thus, *FSH stimulates stem cells proliferation and clonal expansion*. It was intriguing to note how stem cells story which was main focus of our lab research gradually got intertwined with FSH-FSHR biology. Further experiments revealed that when sheep OSE cell smears (Fig. 5) were treated in vitro with FSH, greater numbers of germ cell nests were observed compared to untreated controls after 15 h (Fig. 5B compared to C). Thus, it was clearly evident that FSH stimulated the stem cells to undergo proliferation and clonal expansion. qRT-PCR studies were undertaken to further analyze change in Fshr-1 and Fshr-3 expression after 3 h and 15 h in these cultures in 3 different experiments (Fig. 6).

**Fig. 7:**
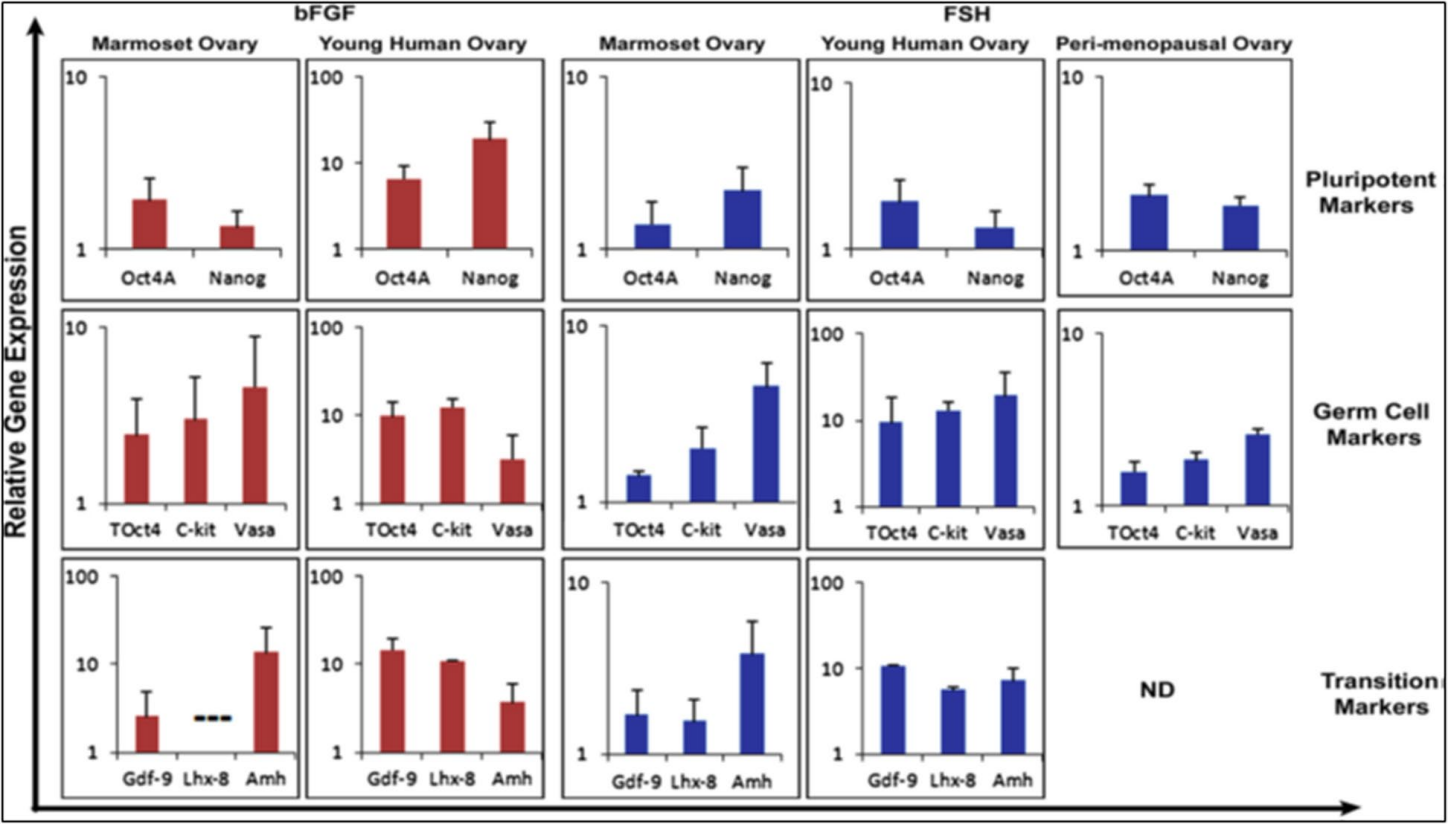
Effect of bFGF (100 ng/ml) and FSH (0.5 IU/ml) treatment on stem cells (Oct-4A, Nanog), germ cells (Oct-4, c-Kit, Vasa) and transition (Gdf9, Lhx-8, Amh) specific transcripts in ovarian cortical tissue culture of marmoset and human ovarian tissue [37]

Later on, Parte et al. [37] studied the effect of bFGF and FSH on human ovarian cortical tissue biopsies in culture (Fig. 7). As evident, both treatments resulted in increased expression of pluripotent (Oct-4A, Nanog), germ cells (tOCt-4, c-Kit, Vasa) and transition (Gdf9, Lhx-8, Amh) specific transcripts. This was again suggestive of stimulatory effect of FSH on ovarian stem cells.

Other groups have also reported FSHR isoforms in mammalian ovaries, human ovarian cancer tissue and cancer cell lines. Sullivan et al. [38] reported that Fshr3 is the pre-dominant isoform in different types (small, medium, large and preovulatory) of follicles in sheep ovaries. Gerasimova et al. [39] detected FSHR isoforms in 35% of cases associated with abnormal ovarian response. Zhou et al. [40] cloned FSHR2 (with deletion of exon 10 and inclusion of 2 small exons after exon 9) and FSHR3 (exon 10 is deleted, had exons 1–6) isoforms in human OSE cells obtained from a patient who underwent surgery because of a cyst. These isoforms were also detected in human follicular fluid of 30 patients undergoing IVF. Perales-Puchalt et al. [41] reported FSHR expression on aggressive ovarian carcinoma samples. Mamas et al. [42] reported that alternately spliced variants of FSHR may be associated with poor or high response to exogenous FSH. Karakaya et al. [43] showed presence of FSHR isoforms in women who were poor responders to FSH treatment. None of the splice variants could initiate cAMP signaling despite high FSH doses. Song et al. [44] studied FSH induced proliferation of epithelial ovarian cancer cells is not mediated via cAMP suggesting a possible involvement of Fshr-3 rather than the canonical Fshr-1 by activating sphingosine kinase.

**Fig. 8:**
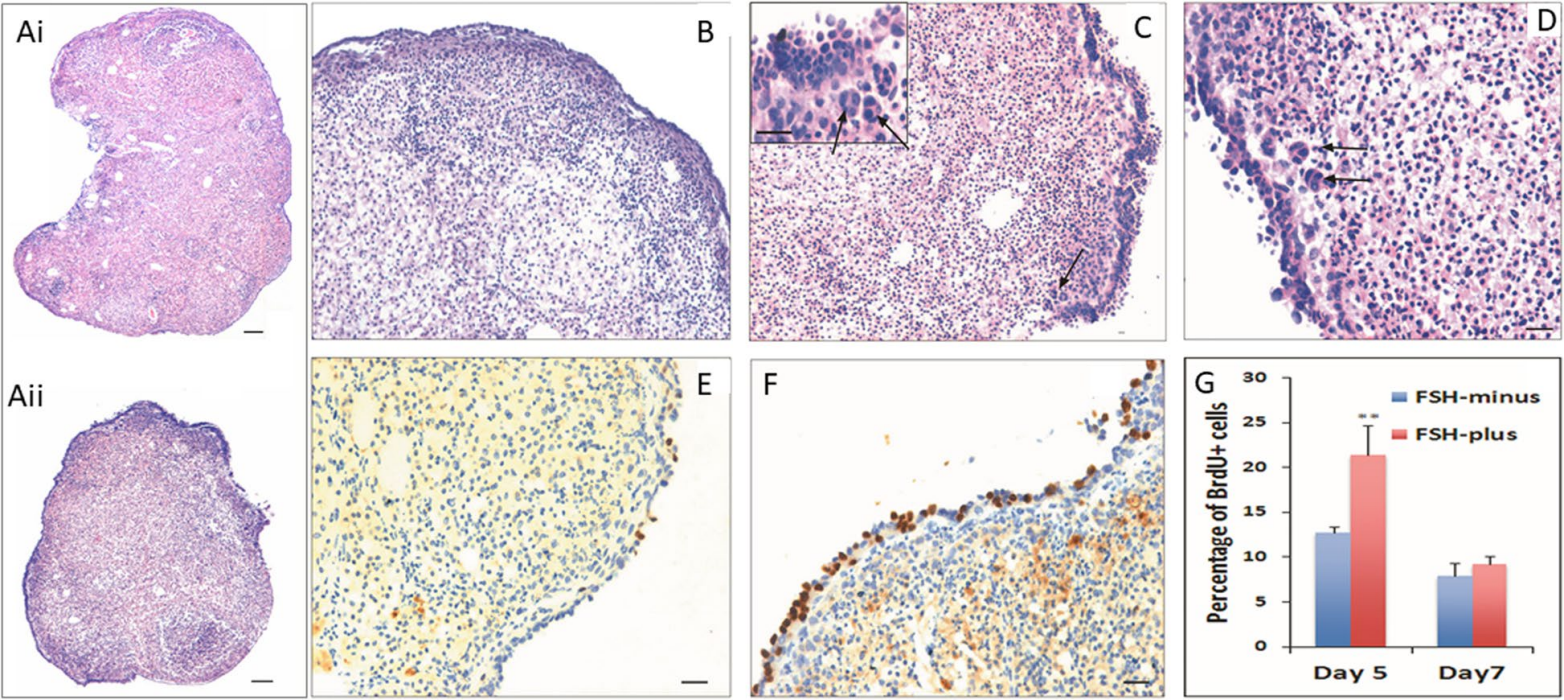
Effect of FSH on chemoablated mouse ovary. Right panel: H&E-stained sections of intact mice ovaries after 7 days culture in FSH-minus (upper) and FSH-plus 10mIU (lower) group. Note extensive proliferation of OSE cells in FSH-plus group. Left panel, Upper images are H&E stained sections at higher magnification showing multilayered OSE in FSH-plus group. Lower images show increased BrdU uptake in FSH-plus group compared to FSH-minus group. Percent BrdU positive cells were much more in FSH treated group on D5 [25]. Scale: 20 μm

Fshr-3 transcripts have been detected in mice OSE cells and not canonical Fshr-1 [45]. Studies were undertaken on 4 weeks old mice after 30 days of treatment with cyclophosphamide (100 mg/kg body weight for first 2 days) and busulphan (10 mg/kg body weight for 4 days). This treatment resulted in complete loss of follicles but flow cytometry studies showed the presence of VSELs in chemoablated ovaries (Fig. 8) [27]. Treatment of chemoablated mice with PMSG (5 IU) and flow cytometry after 48 h showed further increase in VSELs numbers (control ovaries: 0.02 + 0.01%; chemoablated ovaries: 0.03 + 0.02%; PMSG treatment: 0.08 + 0.03%). The chemoablated ovaries were removed and cultured in vitro with and without FSH (5mIU) for 7 days and later studied for the effect of FSH on stem cell markers and proliferation was marked by studying BrdU uptake. An increased expression of stem/progenitor cells (*Oct-4A, Sca-1, Oct-4, Dazl)* was observed along with proliferation in OSE evidenced by BrdU uptake. This was accompanied by the formation of germ cell nests (positive for BrdU, MVH and SSEA-1) and expression of *Scp3* which was not detected in chemoablated ovaries without FSH treatment [27].

To conclude, stem cells residing in the mouse, rabbit, marmoset, sheep and human ovarian surface epithelium express FSHR. Besides acting on the granulosa cells, FSH acts directly on the ovarian stem cells and stimulates them to undergo proliferation, asymmetrical, symmetrical divisions and clonal expansion to form germ cell nests and this action is mediated via Fshr3 in mice, sheep and human ovaries. *This is entirely a new and novel FSH-FSHR biology within the mammalian ovaries and delineated by our group for the first time.* FSHR are expressed on the OSE cells, to be more precise on the stem cells that reside in the OSE whereas the epithelial cells remain distinctly negative. Since cancer involves selective expansion of stem cells, FSHR has been reported on ovarian cancer stem cells. FSH acts via Fshr-3 on the stem cells via ERK/MAPK to bring about proliferation of stem cells.

FORKO mice discussed above [17] developed ovarian cancer despite absence of ovulation is intriguing. Incessant ovulation and repeated damage to the OSE possibly does not initiate cancer. VSELs express FSHR and are easily mobilized and initiate ovarian cancer in presence of increased FSH thus supporting Gonadotropin theory of ovarian cancer as we discussed earlier [19]. However, direct evidence to support the concept that although FORKO mice lack FSHR, VSELs expressing FSHR are mobilized from other tissues to the ovaries and initiate cancer remains to be generated. Virant-Klun’s group has reported increased numbers of VSELs in clinical samples of ovarian cancer (33–35).

### Testicular stem cells

FSHR isoforms have been reported in sheep [46] and humans [39, 47] testes as well as on human testicular germ cell tumors [48, 49]. Similar to ovaries, pluripotent VSELs exist along with the spermatogonial stem cells (SSCs) in the adult human [50] and mice [51, 52] testes. Testicular VSELs are relatively quiescent similar to ovarian VSELs and survive chemoablation in mice [51, 52] and various oncotherapy regimens in human testes [53, 54].

**Fig. 9:**
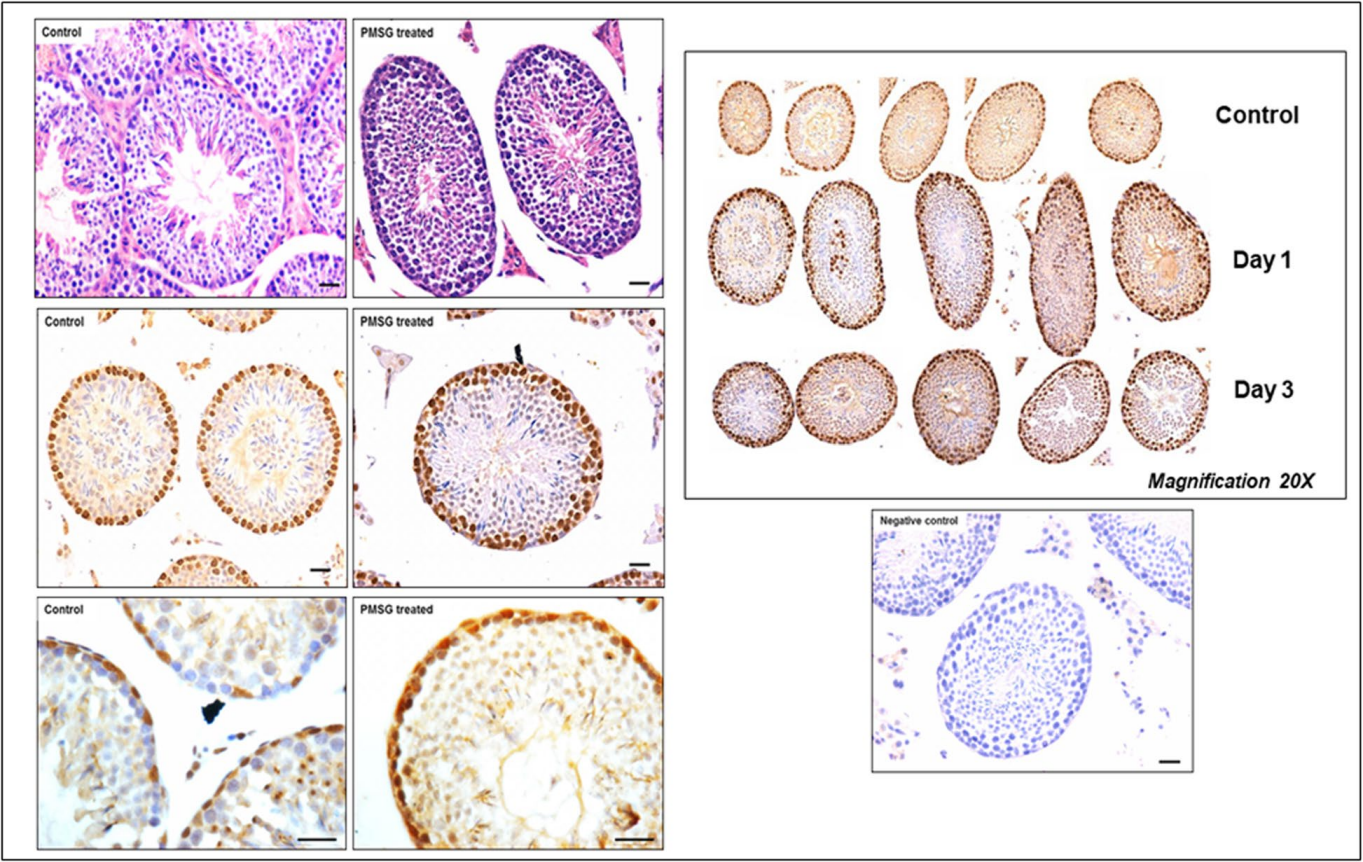
Mice treated with PMSG (10 IU) showed increased numbers of germ cells in H&E-stained sections. This was confirmed by increased PCNA and OCT-4 expression. PCNA expression was studied after 24 and 72 h and increased numbers of germ cells was clearly evident [52]. Scale: 20 μm

**Fig. 10:**
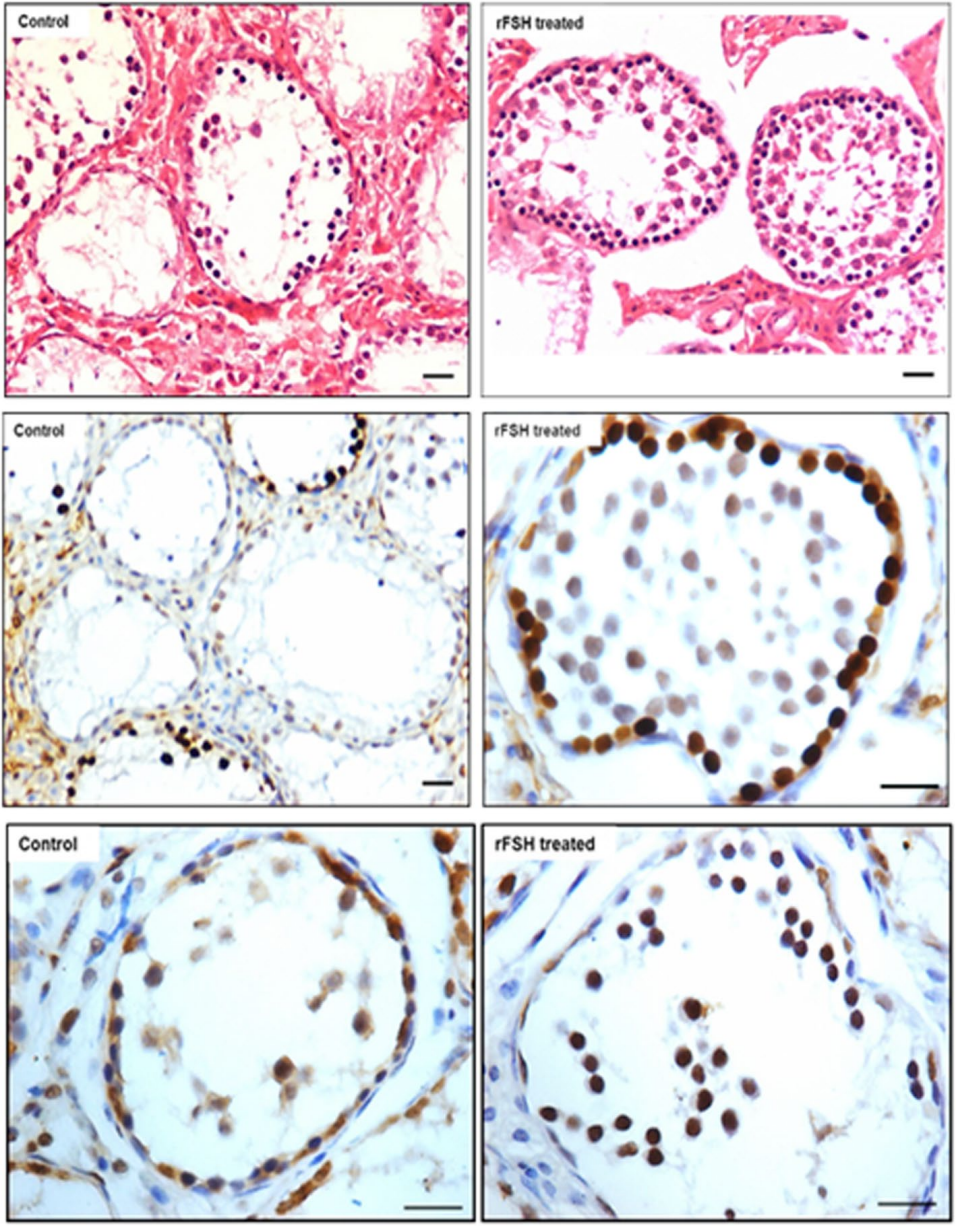
Mice were treated with busulphan (25 mg/Kg) and one month later treated with FSH (10 IU) resulted in increased numbers of germ cells.as evident in H&E stained sections associated with increased expression of PCNA and OCT-4 [52]. Scale: 20 μm

We have recently reported a simple and robust protocol to enrich both the populations of stem cells from mice testes along with their detailed characterization [55]. FSHR expression was studied in normal testes and after 30 days of busulphan treatment (25 mg/Kg) with and without FSH treatment (10 IU per day for 2 days) and PMSG (10 IU per day for 2 days) [52]. VSELs survived chemotherapy and increased in numbers after FSH treatment and this was also confirmed by flow cytometry [52]. Men with idiopathic infertility stand to benefit by FSH treatment as suggested by Simoni’s group [3] probably because FSH directly acts on the tissue-resident stem cells and promotes spermatogenesis. FSHR expression was studied on adult mouse testicular stem cells by immuno-fluorescence using two different commercial antibodies (Abcam, Santacruz), by Western blot, in situ hybridization using oligoprobes specific for Fshr1 and Fshr3 and by qRT-PCR. Fshr-1 and Fshr-3 expression was also studied in mice testes neonatally exposed to endocrine disruption with estradiol (20 μg/pup/ day on days 5–7) and diethylstilbestrol (2 μg/pup/day on days 1–5) [55]. Twenty-four hours of treatment to both intact (PMSG 10 IU, Fig. 9) and chemoablated (4 weeks after busulphan 25 mg/kg, FSH 10 IU, Fig. 10) mice testes were studied [52]. FSH exerted a distinct effect on the stem/progenitor cells confirmed by increased expression of PCNA and OCT-4.

**Fig. 11:**
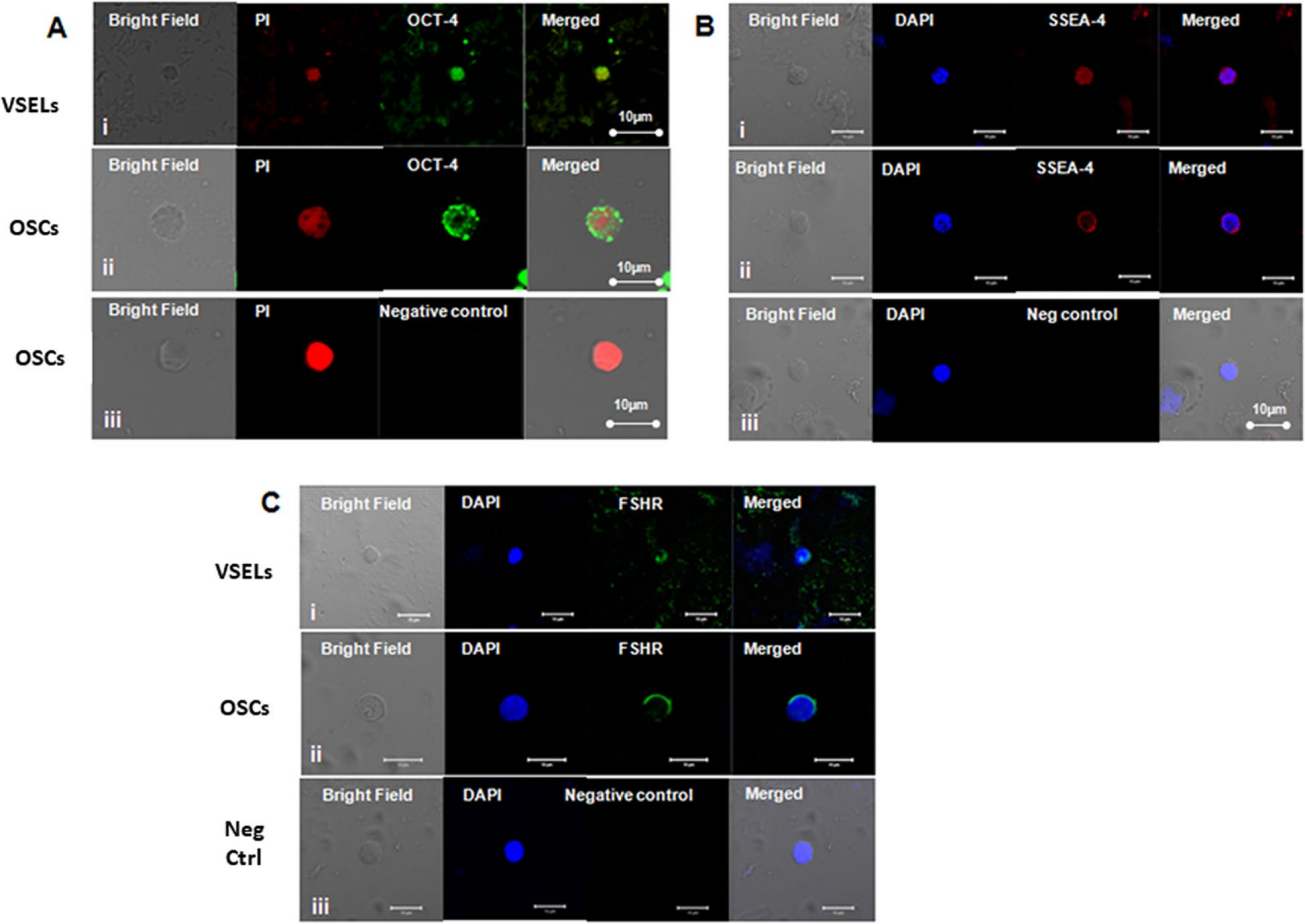
Follicle-stimulating hormone receptor (FSHR) expression on testicular stem cells. **A.** FSHR expression on the stem cells of different sizes and even on dividing cells. FSHR antibody was from ABCAM and raised against N-terminal extracellular domain region (ab150557). Negative controls were with omission of primary antibody and blocking with immunizing peptide was also used to confirm specificity of FSHR expression. **B.** FSHR expression using Santacruz antibody (sc-13,935). **C.** Co-expression of FSHR with OCT-4 and SCA-1 [52]. Scale: 10 μm

Testicular stem cells smears were prepared from chemoablated testes. Fig. 11 shows differential expression of OCT-4 in testicular stem cells, being nuclear in the pluripotent VSELs (A & B) and cytoplasmic in SSCs (C & D). SCA-1 positive stem cells co-expressed OCT-4 (F) and also SCA-1 positive stem cells co-expressed FSHR [52]. These stem cells also express ERα and Erβ and thus are directly modulated by FSH and are vulnerable to endocrine disrupting chemicals which are mostly synthetic estrogens [55]. FSHR expression was observed on testicular stem cells using two different commercially available antibodies (Abcam, Santacruz, Fig. 10). Besides omission of primary antibody as negative control, we also used immunizing peptide to confirm specificity of FSHR expression (Fig. 11). Effect of treatment with FSH (10 IU) for 24 h was studied on the stem cells in normal and chemoablated testes.

**Fig. 12:**
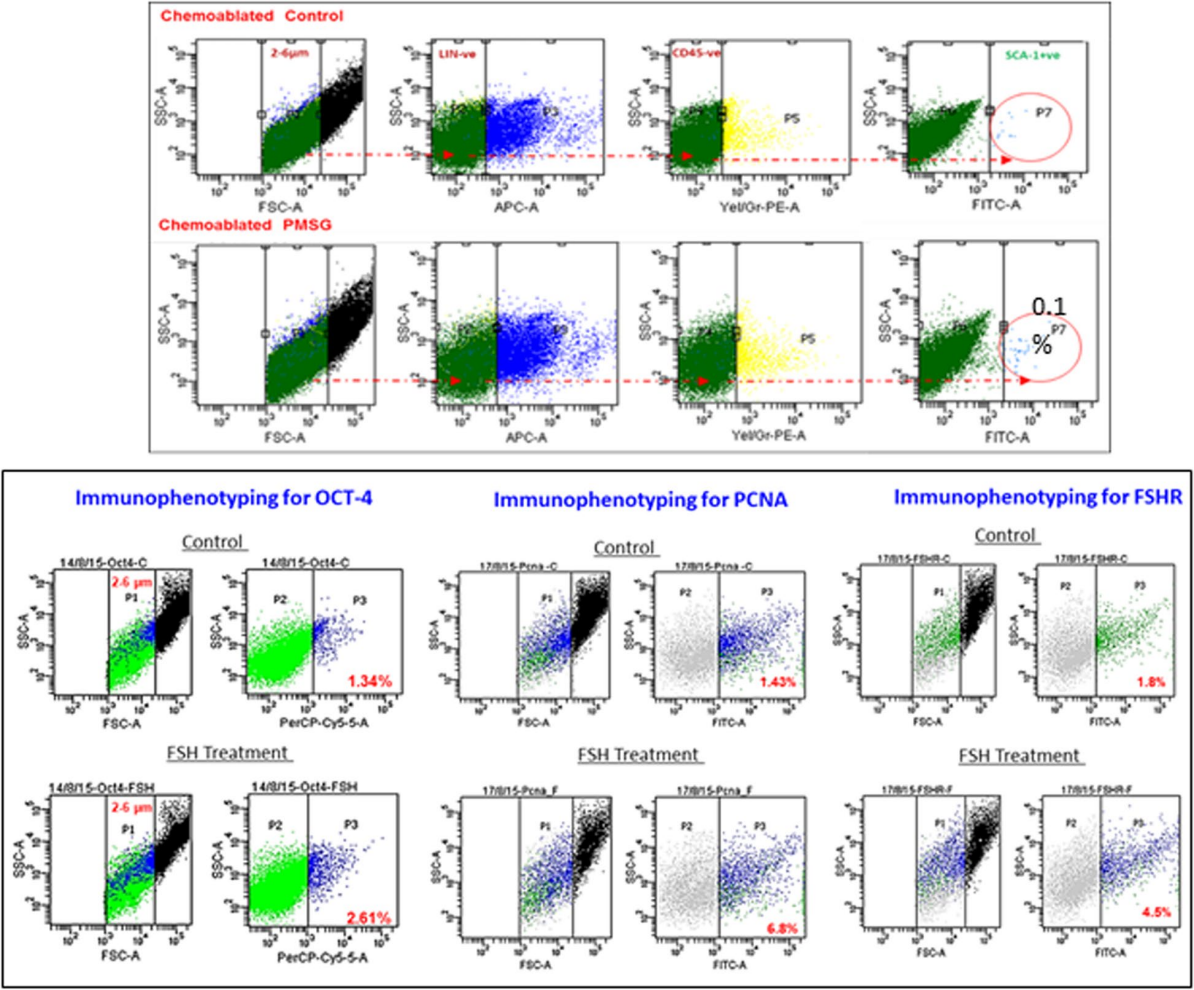
Upper panel: Flow cytometry studies to enumerate very small embryonic-like stem cells (VSELs) in chemoablated testis with and without FSH treatment (10 IU). VSELs were studied as small-sized (2–6 μm), LIN−/CD45−/SCA-1+ cells. VSELs survived in chemoablated testes (0.045 + 0.008% of total cells) and further increased after FSH treatment (0.1 + 0.03%) [51]. Lower panel: Immuno-phenotyping studies for OCT-4, PCNA and FSHR positive cells in chemoablated testes with and without rFSH (10 IU) treatment. FSH resulted in increased numbers of OCT-4 (from 1.34 to 2.61%), PCNA (from 1.43 to 6.8%) and FSHR (from 1.8 to 4.5%) positive stem cells. FSH exerts a direct stimulatory effect on testicular OCT-4 positive stem cells since they express FSHR [52]

As shown in Fig. 12 (upper panel), VSELs in the size range of 2–6 μm with a surface phenotype of LIN-CD45-SCA-1+ were detected in chemoablated testes and their numbers increased from 1.8 to 4.5% upon treating chemoablated mice with FSH. Immuno-phenotyping studies were also undertaken to study OCT-4, PCNA and FSHR in normal and chemoablated testes after treatment with rFSH (10 IU). Lower panel shows rFSH (10 IU) treatment effects on stem cells in chemoablated testes which led to increased numbers of OCT-4 positive stem cells which underwent proliferation (PCNA positive cells increased) and led to increased numbers of FSHR expressing stem/progenitor cells. Thus, similar to in ovaries, in testes also FSH stimulates tissue-resident stem cells.

**Fig. 13:**
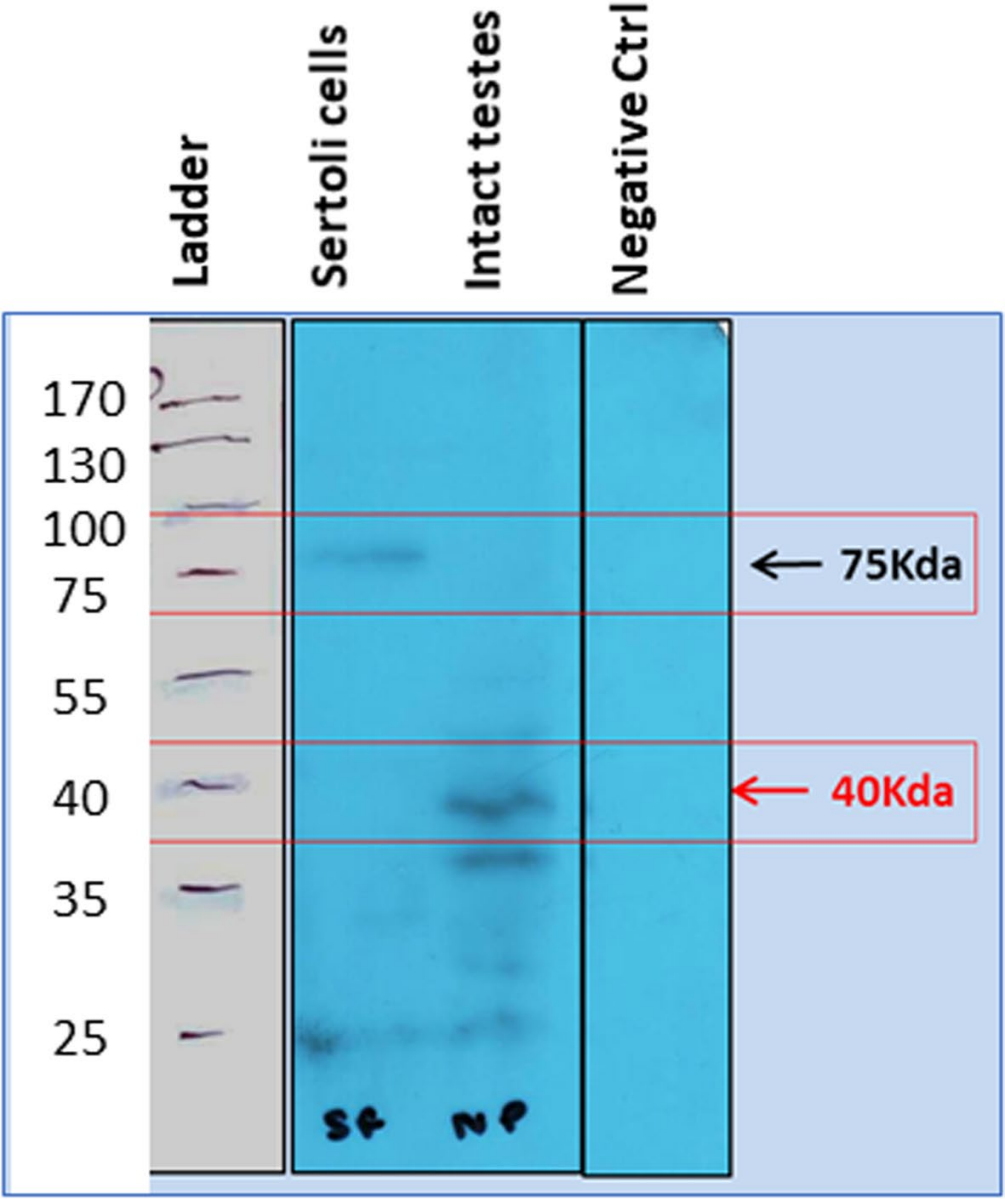
Western blotting for FSHR on FSH treated Sertoli cells and intact testes. Note presence of multiple isoforms after FSH treatment although Sertoli cells only express canonical FSHR-1. **A**. Protein ladder, **B**. Sertoli cells, Intact testes D. Negative control. FSH treatment results in increased expression of FSHR-3 on stem cells

Further Western blotting experiment on proteins extracted from Sertoli cells and intact testes after FSH treatment when hybridized with Abcam FSHR antibody showed presence of 75 kDa band for canonical FSHR-1 in Sertoli cells but in intact testis, two bands of 75 and 40KDa were detected. 75 kDa band was not detected in intact testes after FSH treatment because as such the protein amount may be too less but we clearly observed the band in protein extracted from Sertoli cells. However, FSH treatment affected stem/progenitor cells and FSHR-3 and other isoforms showed increased expression and were detected by Western blot (Fig. 13).

**Fig. 14:**
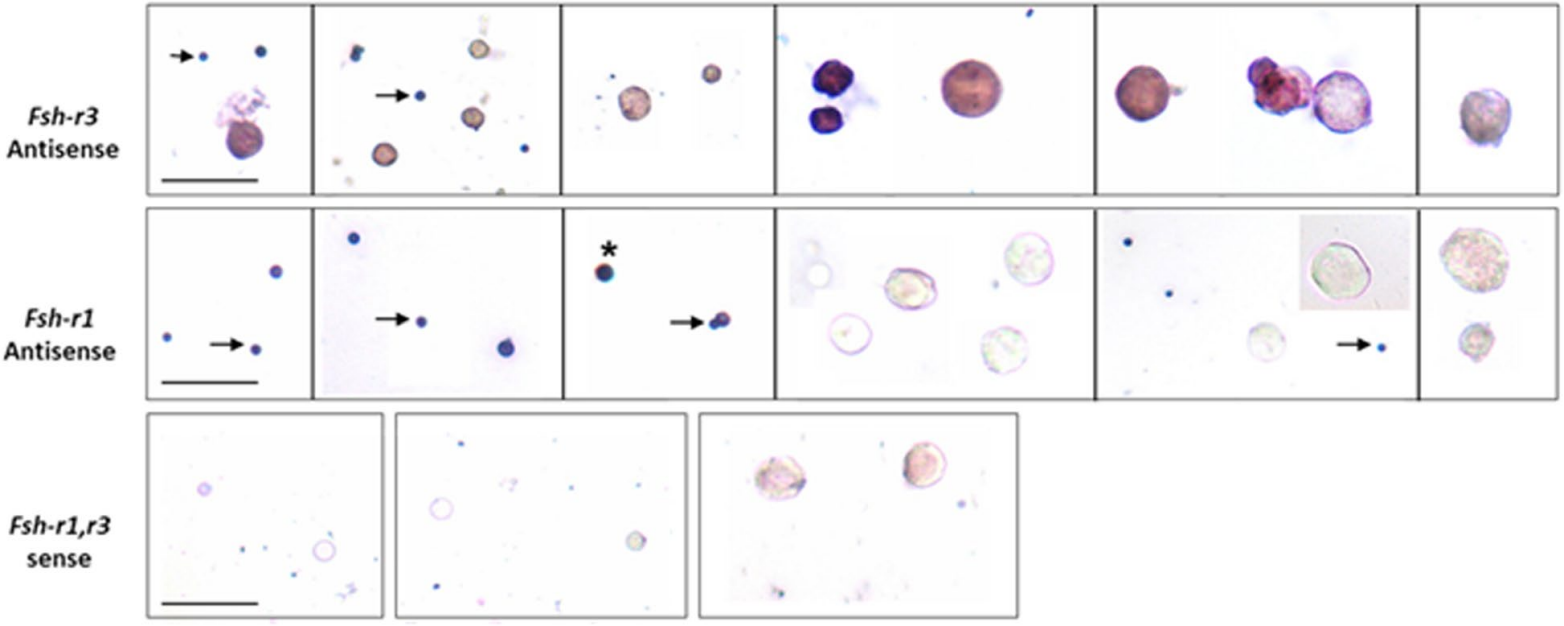
FSHR expression on testicular stem cells. Fshr-1 and Fshr-3 transcripts were studied by in situ hybridization on FSH treated mice testicular cells. As evident Fshr-1 transcript is observed in the nuclei of both VSELs (arrow) and SSCs (asterix). Fshr-3 transcript is localized both in the nuclei and cytoplasm. Presence of Fshr-3 in both cytoplasm and nuclei suggested active involvement of Fshr-3 transcript during FSH action on the stem cells [52]

Expression of FSHR on the stem cells was also confirmed by in situ hybridization using oligoprobes. Both Fshr-1 and Fshr-3 were found expressed on the stem cells. FSH treatment resulted in active transcription of Fshr-3 on dividing stem cells compared to Fshr-1 mRNA (Fig. 14).

**Fig. 15:**
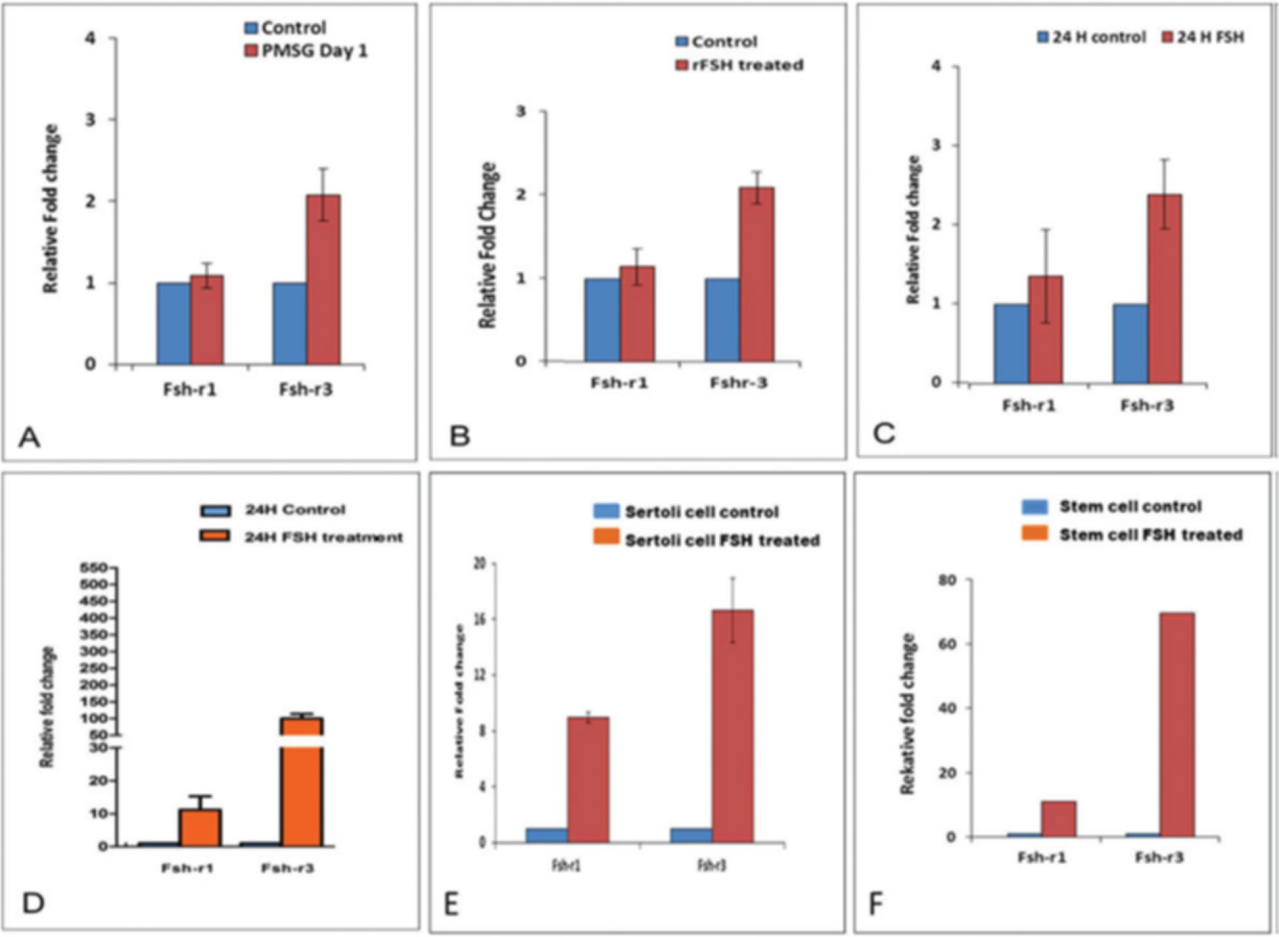
Effect of FSH treatment on alternatively spliced Fshr-1 and Fshr-3 transcripts by qRT-PCR under different experimental conditions. Various treatment groups showed higher upregulation of Fshr-3 compared to canonical Fshr-1, which was minimally regulated. Intact (PMSG 10 IU) (**A**) and chemoablated (rFSH 10 IU) (**B**) testis and total cells isolated from intact (**C**) and chemoablated (**D**) testis. Note > 100-fold increase in Fshr3 in cells in vitro from chemoablated testis. Sertoli cells also respond to FSH treatment, and both Fshr-1 (> 8 fold) and Fshr-3 (> 16 fold) transcripts are upregulated. SCA-1 sorted cells showed > 80 fold increase in Fshr3 compared to minimal increase in Fshr-1. Fshr-3 appears to be the more predominant transcript through which FSH exerts its action on the stem cells [52]

Expression of Fshr1 and Fshr3 were also studied by qRT-PCR in different treatment groups (Fig. 15). It was clearly observed that alternatively spliced Fshr-3 was markedly regulated compared to Fshr-1 in various treatment conditions [52]. Single-cell suspensions (including both Sertoli cells and germ cells) obtained after enzymatic digestion, from both intact and chemoablated testes was cultured in DMEM/F-12 medium containing 10% FBS and antibiotics with and without rFSH (5 IU/mL) for 24 h. Cells were harvested 24 h later for qRT-PCR studies. Sertoli cells were separated by differential plating. For this, testicular cells were plated in 60 mm culture dishes, Sertoli cells get attached whereas the germ cells remain floating and are removed by gentle change in medium. Sertoli cells were allowed to grow to 70% confluence, treated with FSH (5 IU) for 3 h, and then harvested for qRT-PCR studies. Magnetic enrichment of SCA-1-positive cells from chemoablated testes was carried out using FITC conjugated SCA-1 antibody and EasySep kit (Stem Cell Technology, Vancouver, Canada). Stem cell antigen 1 (SCA-1) positive cells were plated in a 35 mm culture dish and incubated overnight with and without FSH (5 IU/mL). Cells isolated from intact and chemoablated (enriched for quiescent VSELs as all actively dividing cells are destroyed by chemotherapy) testes were put in culture and studied for Fshr expression after 24 h in vitro. Note > 100 fold increase in Fshr-3 in cells collected from chemoablated testes upon treatment with PMSG (10 IU). Sertoli cells also responded to FSH treatment and both Fshr-1 (> 8 fold) and Fshr-3 (> 16 fold) transcripts were upregulated. SCA-1 sorted cells showed > 80 fold increase in Fshr-3 compared to minimal increase in Fshr-1. Fshr-3 was the more predominant transcript through which FSH exerts its action on the testicular stem cells and also on Sertoli cells.

**Fig. 16:**
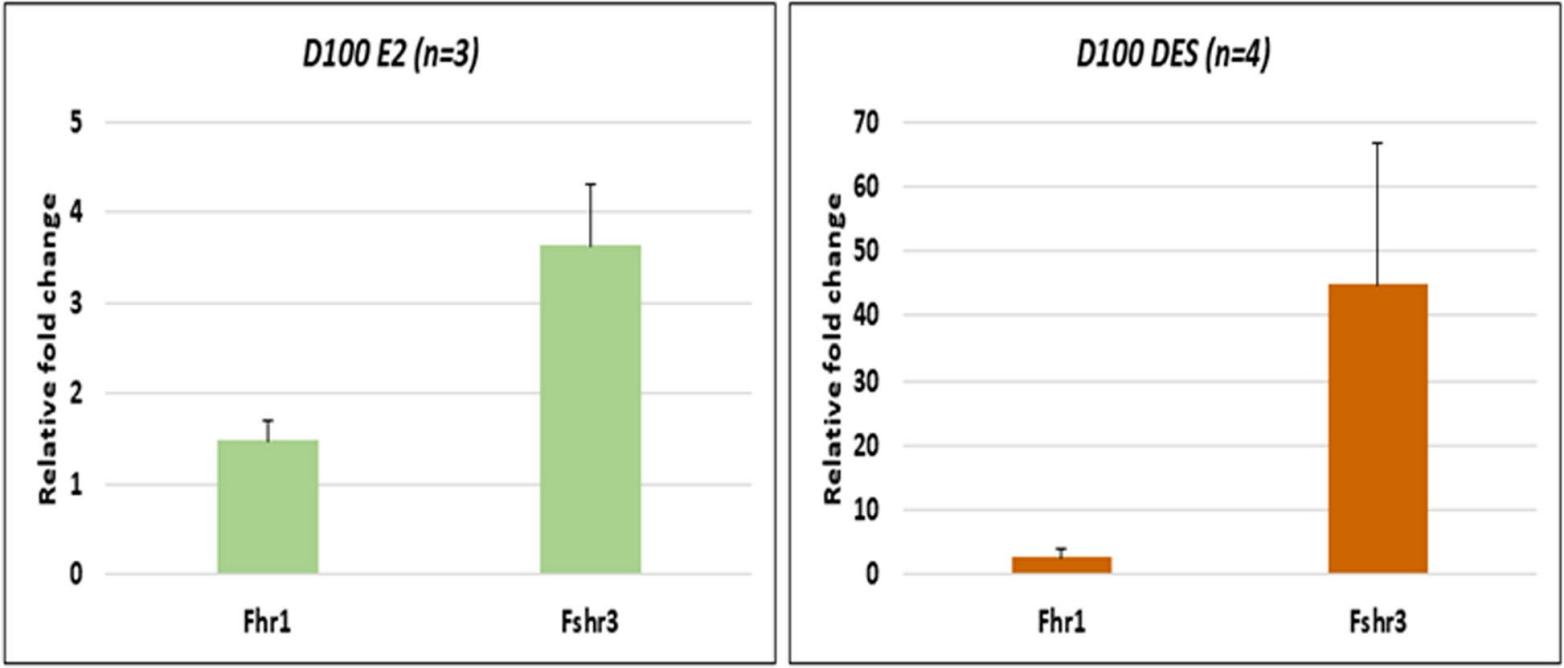
Fshr-1 and Fshr-3 expression in 100 days old testes of mice neonatally exposed to estradiol (E2, 20 μg/pup/day on days 5–7) and diethylstilbestrol (DES, 2 μg/pup/day on days 1–5). Both treatments resulted in increased numbers of stem cells and their blocked differentiation. However, marked increase in Fshr-3 was noted after DES which resulted in testicular cancer-like changes. Cancer occurred due to excessive self-renewal of VSELs which expressed FSHR-3 [unpublished data, related to 56]. Data represents mean + se

In a recent study, mice pups were exposed to estradiol (20 μg/pup/day on days 5–7) or diethylstilbestrol (2 μg/pup/day on days 1–5) and later studies were undertaken on D100 of adult life. Treatment affected the testicular stem cells and resulted in pathologies including disrupted spermatogenesis, reduced sperm counts, infertility and tumor-like changes were observed in DES treated group [56]. Expression of *Fshr1* and *Fshr3* was studied on D100 by qRT-PCR (Fig. 16). Fshr-3 was markedly upregulated after both estradiol and DES treatment whereas canonical Fshr-1 expression remained unaffected. DES treatment resulted in cancer-like changes in the testes accompanied with marked up-regulation of Fshr-3 ranging from 15 to 100 folds [56]. In two samples after DES treatment, > 800 folds increase in Fshr-3 transcripts was observed but was not included in Fig. 15 since the Ct value > 35.

The results compiled above are intriguing since FSHR expression is expected only on the Sertoli cells as per existing understanding [1]. Results suggest a direct action of FSH on the testicular stem cells. Similar to ovaries, in testes also, FSH directly stimulated the stem cells to undergo ACD, SCD and clonal expansion [52, 57]. More than 40 folds increase in Fshr-3 in DES treated testes with cancer-like changes along with increased expression of embryonic markers including Oct-4A (> 10 folds) [56] which is a specific and sensitive marker for testicular cancer [58].

### Uterine stem cells

Uterus is considered as an end organ for the steroid hormones estrogen (E) and progesterone (P) to act. These hormones are secreted by the ovaries under the influence of pituitary gonadotropins and thus it is generally believed that FSH exerts an indirect effect on the uterus. However, few reports have challenged this existing dogma in the field. Increased expression of FSHR was reported in the secretory phase human endometrium compared to proliferative phase [59, 60]. Later FSHR expression was reported in human uterus and placenta. Stilley et al. [61] reported FSHR on the endothelial cells of blood vessels in the endometrium, myometrium, and cervix; endometrial glands of the proliferative and secretory endometrium; cervical glands and the cervical stroma; and in stromal cells (at low levels) and muscle fibers of the myometrium in non-pregnant women. During pregnancy, placental FSHR was detected by 8–10 weeks of gestation up to term on the endothelial cells in fetal portions of placenta and umbilical cord; epithelial cells of the amnion; decidualized cells surrounding the maternal arteries in the maternal decidua; and the stromal cells and muscle fibers of the myometrium, with particularly strong expression at term. Moreover, genetically modified mice lacking Fshr in fetal portions of the placenta revealed adverse effects on feto-placental development [61]. Robin et al. [62] reported FSHR expression on endometriotic tissue by immunohistochemistry studies. Ponikwicka-Tyszko et al. [63] reported functional expression of FSHR in endometriotic lesions. FSHR is reported in uterine myomas also [64]. This body of literature is looked at with disbelief by others [9, 11] who are perplexed by this extragonadal expression of FSHR. But results are true, only more efforts are required to interpret and understand them.

Our group has reported two populations of stem cells including VSELs and slightly bigger EnSCs in adult mouse uterus and recently published a robust protocol to enrich them from mice uterus [65]. VSELs survive in the atrophied uterus of bilaterally ovariectomized mice and effects of treatment with physiological levels of hormones were reported on the uterine VSELs/EnSCs [66]. Also, effects of treatment with estradiol, progesterone and FSH on both myometrium [67] and endometrium [68] were reported but here discuss the effects of only FSH treatment (5 IU/day for 7 days) on both the myometrium and endometrium.

**Fig. 17:**
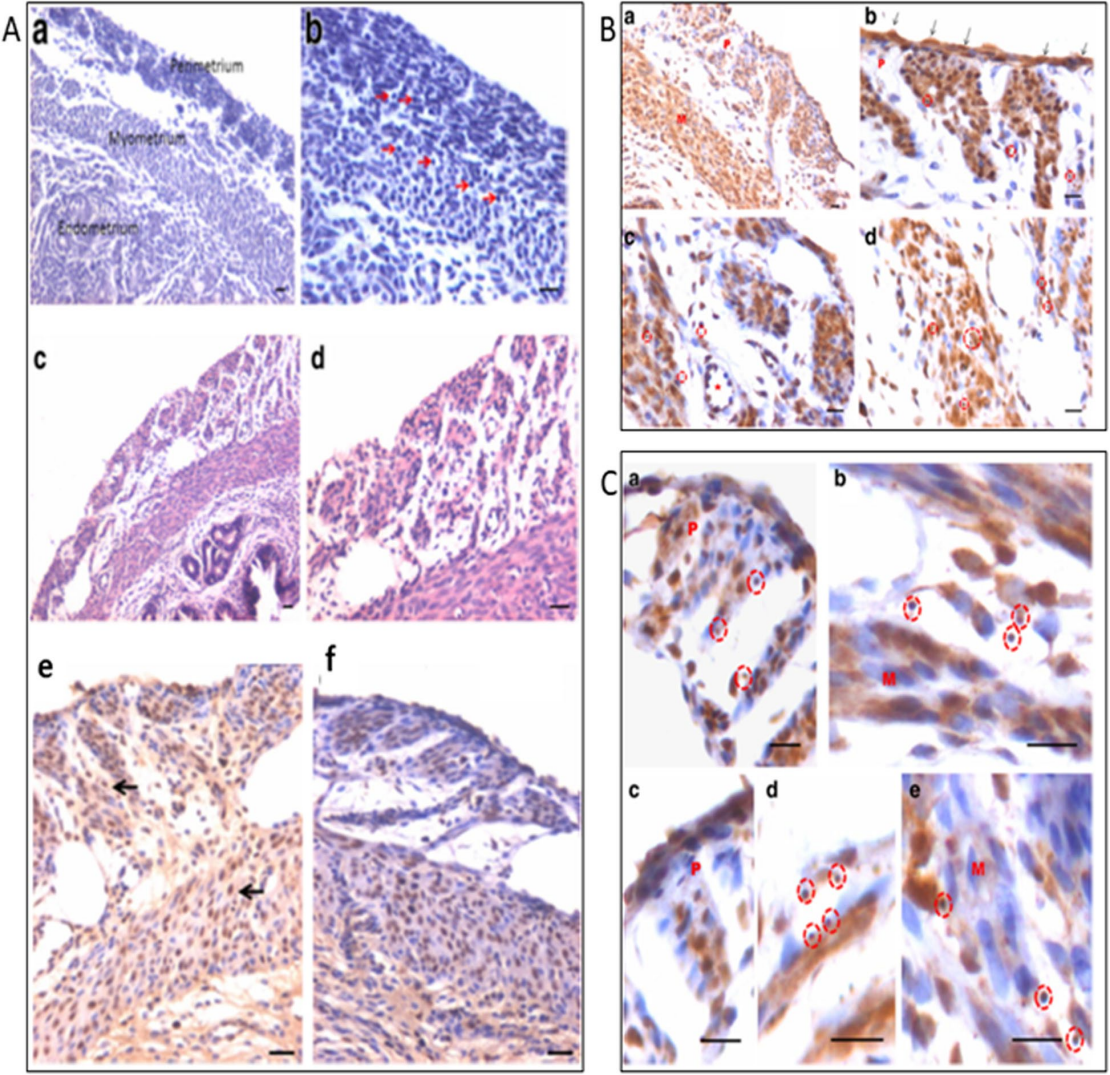
Uterine myometrium in adult mice after 5 days of FSH treatment to bilaterally ovariectomized mice. **A** a&b: Myometrium of bilaterally ovariectomized mice with small spherical stem cells (red arrows) c, d: After 5 days of FSH treatment e,f: PCNA expression in FSH treated group. **B** a-d: OCT-4 expression in FSH treated myometrial sections. Note presence of small, spherical cells expressing FSHR and OCT-4. **C** a-d: Higher magnification showing small spherical cells with nuclear OCT-4. Note bigger myometrial ‘progenitor’ cells with cytoplasmic OCT-4 [67]. Scale: 20 μm

Myometrium was atrophied after bilateral ovariectomy and both perimetrium and myometrium were clearly visualized in the ovariectomized uterus along with small spherical stem cells (Fig. 16, arrow). FSH treatment stimulated the myometrium and large numbers of cells were visualized which expressed both PCNA as well as OCT-4. At higher magnification, small spherical OCT-4 positive VSELs were clearly visualized in the myometrium along with mesenchymal progenitors with cytoplasmic OCT-4. At higher magnifications, the small spherical cells were clearly visualized (Fig. 17, red broken circle) in both the perimetrium and myometrium along with progenitors with cytoplasmic OCT-4. These findings are intriguing since they demonstrate a direct effect of FSH on the stem/progenitor cells residing in the myometrium.

**Fig. 18:**
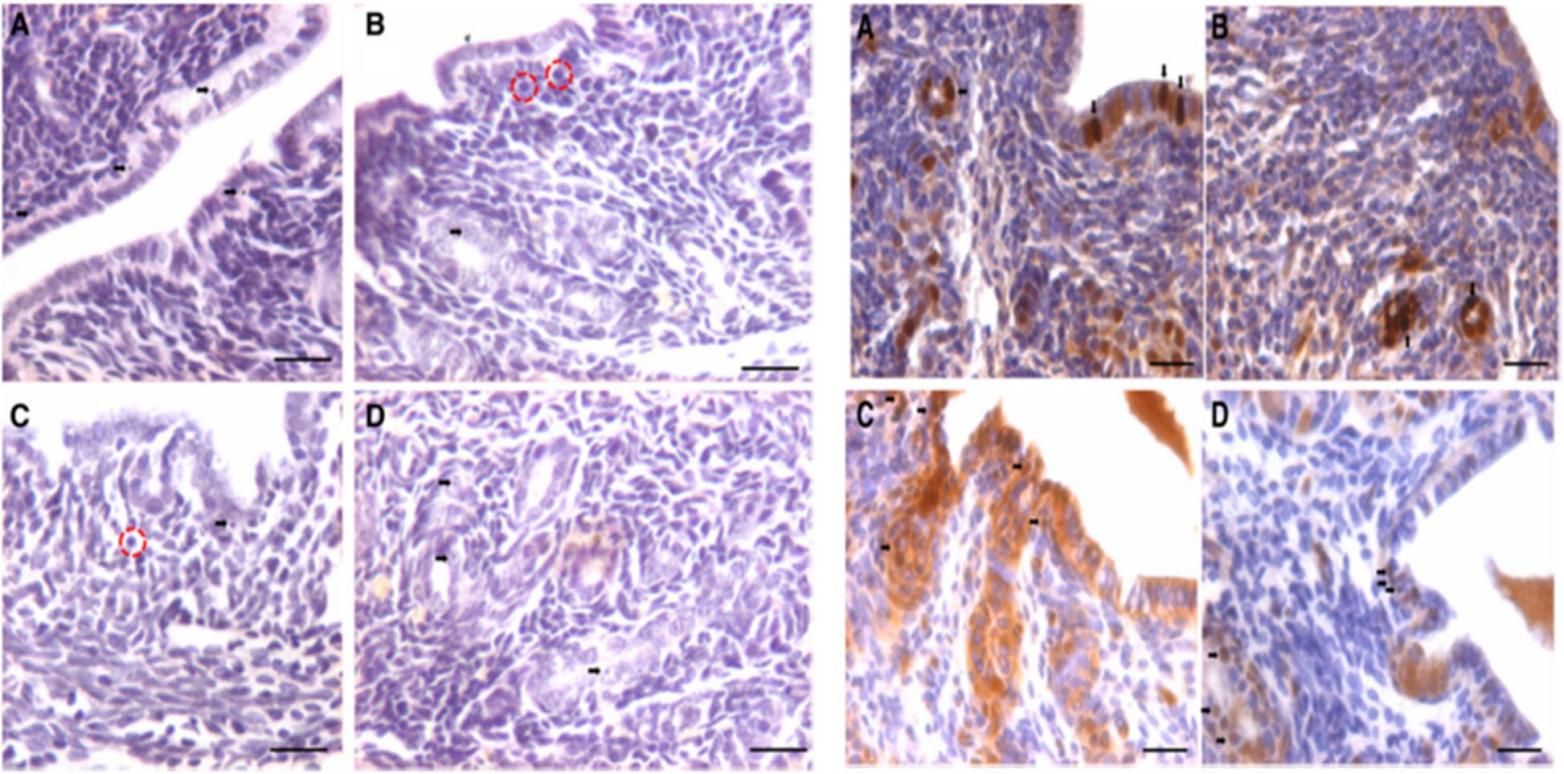
VSELs survive ovariectomy in uterine endometrium. Left panel: Atrophied endometrium after bilateral ovariectomy showed the presence of small spherical cells (VSELs, arrows). Right panel: Minimal PCNA expression was noted in ovariectomized uterine sections in small spherical cells in the epithelial layer (upper panel). OCT-4 expression was noted in both the cytoplasm and nuclei of small spherical cells (lower panel) [68]. Scale: 20 μm

A careful scrutiny of the H&E stained sections of atrophied endometrium (Fig. 18) after bilateral ovariectomy also showed the presence of small spherical cells with dark stained nuclei, which probably are the VSELs that survive ovariectomy. Expression of PCNA and OCT-4 was studied in the atrophied endometrial sections. As evident few cells with distinct nuclear PCNA expression and cells with both nuclear and cytoplasmic OCT-4 were observed.

**Fig. 19:**
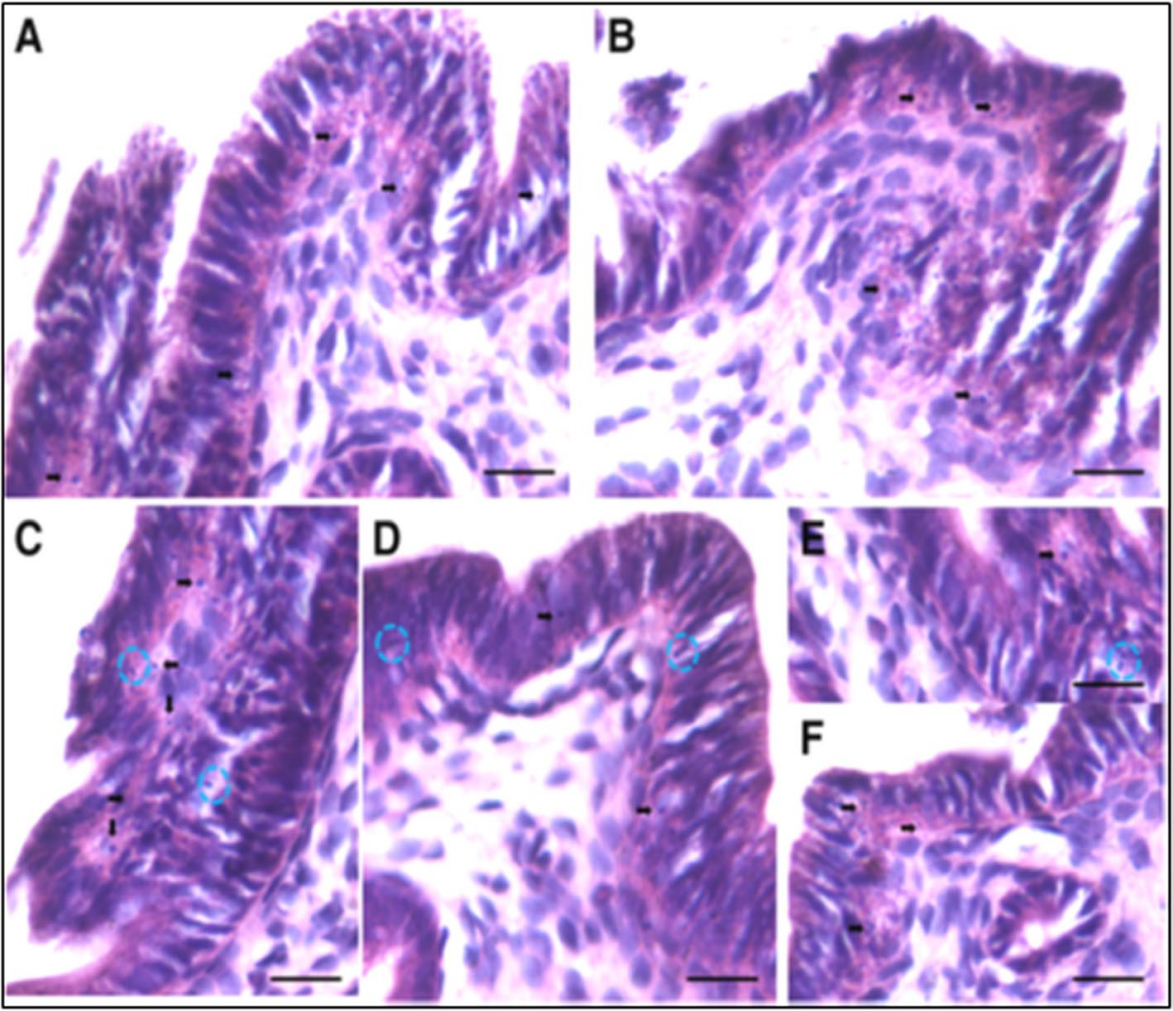
FSH exerts direct effect on the endometrial epithelial cells and the stem cells. FSHR treatment for 5 days exerted a marked effect on the endometrial epithelial cells which showed both hyperplasia and hypertrophy. Note the presence of small spherical cells which are the VSELs cut in different plains of sections. They also appeared to be dividing at few places (broken circles) [68]. Scale: 20 μm

**Fig. 20:**
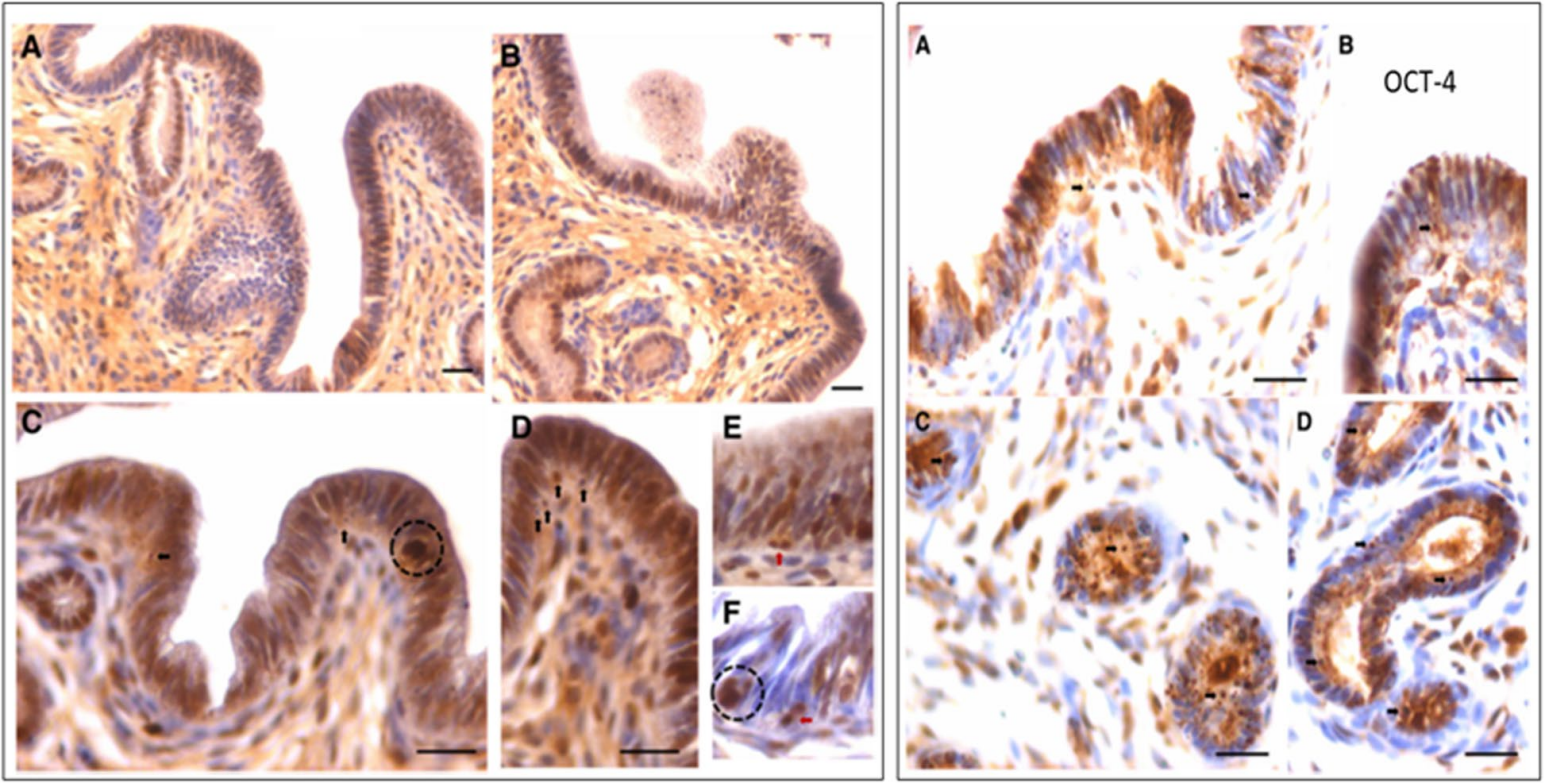
Left panel: Hyperplasia of epithelial cells was confirmed by nuclear expression of PCNA after FSH treatment. Stem cells also expressed PCNA (arrows). Small clusters of cells also expressed PCNA (broken circle in C, F). Right panel: Small spherical cells also expressed nuclear OCT-4 whereas the epithelial cells expressed cytoplasmic OCT-4 [68]. Scale: 20 μm

FSH treatment exerted a marked effect on the endometrial epithelial cells which showed hypertrophy and hyperplasia (Fig. 19). Small spherical VSELs were observed cut in different planes of sections and at places appeared to be dividing (broken circle). Thus, FSH appeared to exert a direct effect on the epithelial cells and on the uterine stem cells. Hyperplasia was confirmed by studying PCNA expression which remained nuclear (Fig. 20). Small clusters of cells and singly existing stem cells were observed positive for PCNA. The small spherical cells also expressed nuclear OCT-4 whereas the epithelial cells expressed cytoplasmic OCT-4. This relationship of nuclear OCT-4 and cytoplasmic OCT-4 in stem and epithelial cells shows the hierarchy amongst various cell type and pluripotent stem cells with nuclear OCT-4 differentiate into epithelial cells with cytoplasmic OCT-4.

**Fig. 21:**
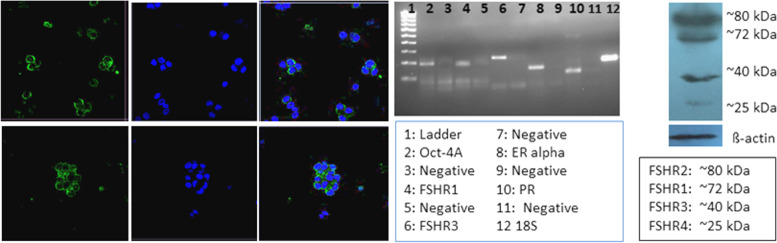
Left panel: FSHR expression on mice uterine stem cells. Right panel: Stem cells in adult mouse uterus express embryonic markers, Fshr1, Fshr3, ER alpha and PR [65]. Western Blot showed presence of 4 alternately spliced FSHR isoforms in adult mouse uterus [68]

Uterine stem cells can be enriched by spinning at 1000 g and visualized after H&E staining [65]. The stem cells have been well characterized, express embryonic markers and ER, PR and FSHR (Fig. 20A). RT-PCR showed presence of pluripotent transcripts Oct-4A along with Fshr1, Fshr3, Erα, PR (Fig. 20B). Surprisingly all the 4 alternately spliced isoforms of FSHR were detected in the uterus by Western blotting (Fig. 21).

**Fig. 22:**
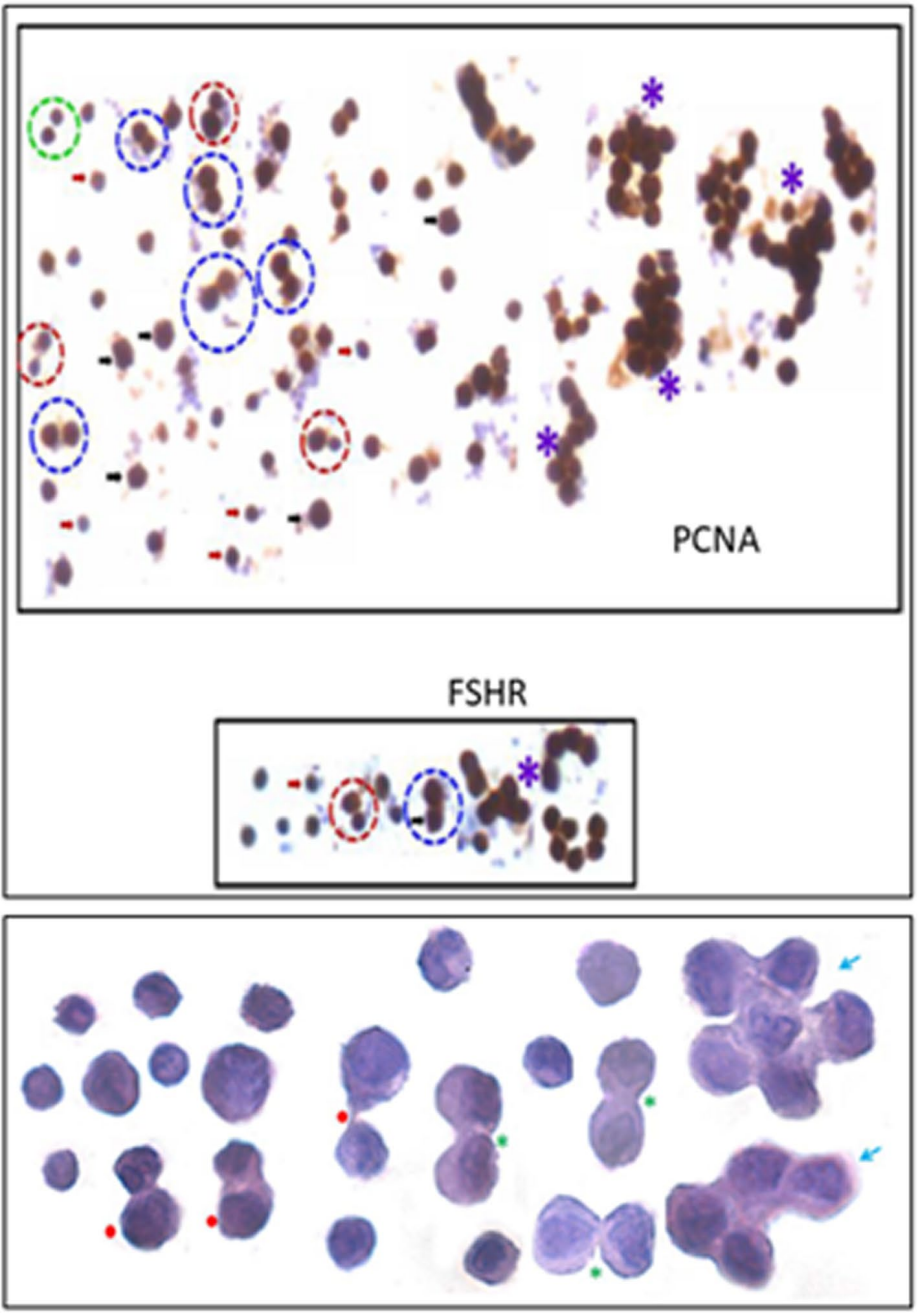
Two populations of stem cells including VSELs (red arrow) and slightly bigger EnSCs (black arrow). Stem cells smears from the uterus showed asymmetrical (red broken circle), symmetrical (blue broken circle) divisions and clonal expansion (purple asterix). These stem cells expressed PCNA and FSHR. Higher magnification clearly showed asymmetrical (red asterix), symmetrical (green asterix) divisions and clonal expansion (blue arrow) [57, 68] Scale: 20 μm

The stem cells enriched from adult uterus were used to prepare smears and studied for expression of PCNA and FSHR (Fig. 22). Two distinct stem cells populations including small VSELs (red arrow) and slightly bigger EnSCs (black arrow) were clearly visualized. The cells undergo asymmetrical (red broken circle), symmetrical (blue broken circle) divisions and clonal expansion (blue asterix). These divisions were also observed at higher magnifications (Fig. 22, bottom panel).

To conclude, two populations of stem cells exist in adult mouse uterus which express FSHR and FSH exerts direct effect on the uterine stem cells similar to in the ovaries and testes. Early differentiating progenitors express embryonic markers as well as FSHR. We have recently shown that neonatal endocrine disruption affects uterine stem cells differentiation and progenitors increase in numbers associated with increased FSHR expression [69]. Moreover, various pathologies like endometriosis, fibroids and endometrial cancers express increased FSHR because they arise due to affected differentiation and selective expansion of FSHR and OCT-4 positive stem/progenitor cells.

### Bone marrow stem cells

Hematopoietic system also harbors two populations of stem cells including VSELs and hematopoietic stem cells (HSCs) [7, 70], although there is no consensus as yet. Evidence has accumulated over time that hematopoietic stem cells share several markers with the germline and that treatment with prolactin, androgens, and estrogens stimulate hematopoiesis. Ratajczak’s group [71] for the first-time reported expression of functional FSH and LH receptors on hematopoietic stem cells and VSELs and also on embryonic stem cells and teratocarcinoma cells [12]. Our group also reported expression of functional FSHR on bone marrow VSELs and HSCs in adult mice [72]. Abdelbaset-Ismail et al. [73] reported expression of receptors at both mRNA and protein levels, for FSH, luteinizing hormone, prolactin, progesterone, estrogen, and androgen on hematopoietic stem cells including VSELs isolated from human umbilical cord blood and peripheral blood. Sex hormones in vitro enhanced clonogenic growth of the hematopoietic stem cells. Zbucka-Kretowska et al. [74] reported statistically significant mobilization of VSELs and HSCs (and not endothelial progenitors) into the peripheral blood of 15 female patients undergoing FSH therapy in the infertility clinics. This body of literature suggests developmental origin of VSELs and HSCs from germ lineage. Receptors for pituitary and ovarian hormones are also reported on leukemic cell lines and blasts. Virant-Klun [75] discussed the developmental link between germ lineage and hematopoiesis in humans and later on a review was published discussing why the hematopoietic stem cells are so ‘sexy’ [76].

**Fig. 23:**
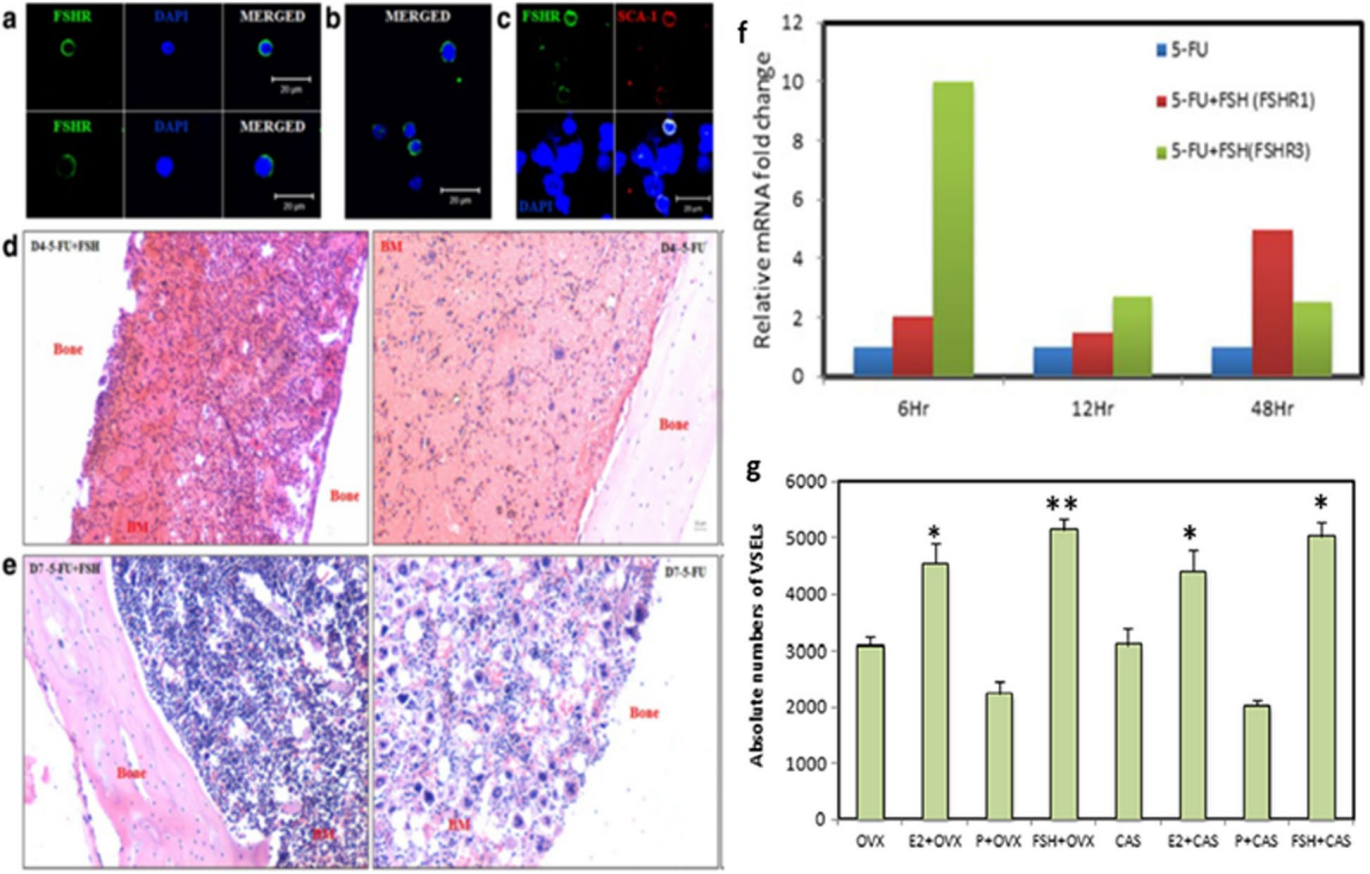
(a-b) Mouse bone marrow VSELs and HSCs also express FSHR. (c) Also note co-expression of a stem cell marker SCA-1 and FSHR. (d-e) Mouse decalcified bone sections after chemoablation (5-flurouracil, 150 mg/kg) to study the effect of FSH on resumption of hematopoiesis. Left panel in d & e are FSH treatment to chemoablated mice on D4 and D7 compared to no treatment in the right panel. FSH enhanced hematopoiesis by almost 72 h by exerting a direct action on VSELs [72]. (f) As evident in 5-FU treated mouse bone marrow, 10-fold increase in FSHR3 was observed 6 h after FSH (5 IU) treatment [56]. (g) Ovariectomized and castrated mice were treated with Estradiol (E2, 2 μg/day), progesterone (P, 1 mg/Kg/day) and FSH (5 IU per day) for 7 days and absolute numbers of VSELs were enumerated. FSH exerted a direct action and VSELs numbers were increased similar to after E2 treatment whereas P did not exert any effect [77]. Scale: 20 μm

Functional studies were reported in vivo by our group on adult mice bone marrow showing a direct effect of FSH on hematopoiesis [72]. Treatment with 5 fluorouracil (5-FU, 150 mg/Kg) depletes the bone marrow of mature blood cells. These chemoablated mice were studied with and without FSH (5 IU) treatment on days 2, 4 and 10 after chemotherapy. FSH treatment enhanced hematopoietic recovery by at least 72 h in 5-FU-treated mice and this action of FSH was mediated via Fshr3 and almost 10-fold increase in Fshr-3 was observed by 6 h of FSH treatment [72]. In another study, Ganguly et al. [77] treated ovariectomized and castrated mice with E, P & FSH and as evident FSH was observed to exert profound effects and resulted in increased numbers of VSELs by flow cytometry studies (Fig. 23).

**Fig. 24:**
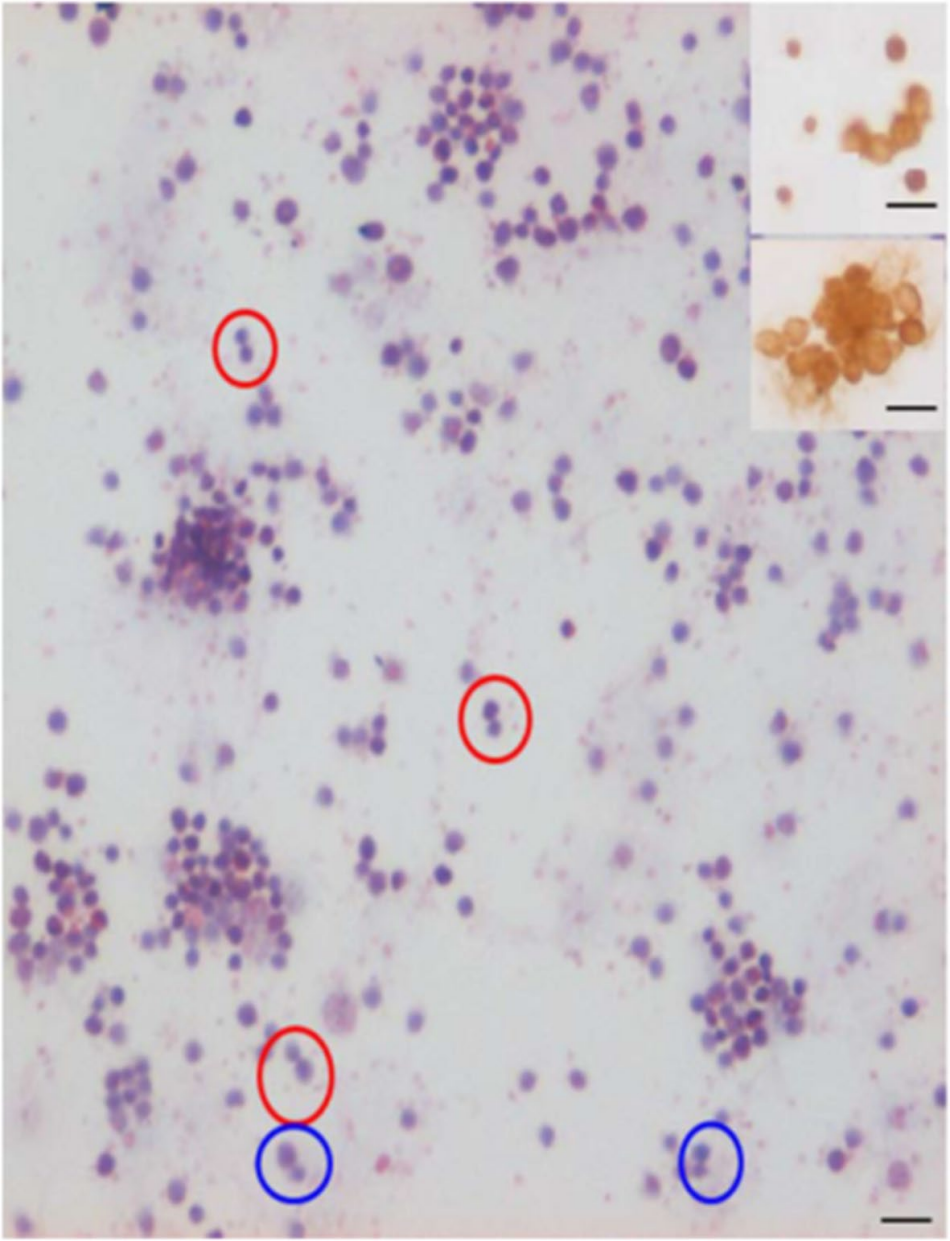
A**.** H&E stained bone marrow stem cell smears prepared from a chemoablated mice (5-FU, 150 mg/Kg) after treatment on Days 4 & 5 with FSH (5 IU) and sacrificed on Day 6. One could see various types of cell divisions including ACD (blue circle), SCD (red circle) and clonal expansion. Insert shows that the cells enriched by 5-FU in the bone marrow express OCT-4 [57]. Scale: 20 μm

Effect of FSH treatment was also studied on stem cells in bone marrow cell smears. For this 5-FU treated mice were further treated with FSH (5 IU) on days 4 & 5 and sacrificed on Day 6. Bone marrow was flushed out and used to make cell smears (Fig. 24). One could easily visualize asymmetrical, symmetrical divisions and clonal expansion in the stem cells compartment [56].

Similarly, VSELs in mouse prostate are regulated by FSH and only Fshr3 is expressed in adult prostate and not the canonical Fshr1 (unpublished data by our group). These results are in contradiction with recent scRNAseq study [78] which negated presence of stem cells in mice and human prostate. We had pointed out reasons for their inability to detect stem cells [79, 80].

*In view of the research findings compiled in this review, it becomes imperative to appreciate a wider landscape for FSH action which is so far limited to Sertoli cells in testes and Granulosa cells in ovaries.*

## Data Availability

It should be present and appropriate for data policy associated with the journal (stated in the submission guidelines). Not applicable.
